# Slippery bioactive nanoemulsion-infused and nitric oxide-releasing surfaces for anti-fouling biomedical applications

**DOI:** 10.1039/d6bm00767h

**Published:** 2026-08-04

**Authors:** Grace H. Nguyen, Everett Grizzle, Elizabeth J. Brisbois

**Affiliations:** a School of Chemical, Materials, and Biomedical Engineering, University of Georgia 302 East Campus Rd. Athens GA 30602 USA ejbrisbois@uga.edu +1 706-542-1243

## Abstract

Indwelling medical devices are highly susceptible to infection and thrombosis, two major complications arising from bacterial adhesion, protein deposition, and platelet aggregation on device surfaces. To address these challenges, a multifunctional surface that integrates slippery nanoemulsion (NE) infusion with dual bioactive release of nitric oxide (NO) and eugenol (EG) was developed. *N*-hexadecane-based NEs, with or without eugenol incorporation, were infused into *S*-nitroso-*N*-acetylpenicillamine (SNAP)-impregnated polydimethylsiloxane (PDMS) to create materials featuring a stable lubricant reservoir that provides anti-fouling properties and is combined with an active antimicrobial and antithrombotic agent. The combined material SNAP-2%EG exhibited low sliding angles (<20°) while maintaining near-physiological NO release for 24 h. The SNAP-2%EG did not elicit cytotoxic effects on mouse fibroblast cells, with relative cell viability >70%, while significantly reducing the adhesion and viability of *Staphylococcus aureus* and *Escherichia coli* by 2.95 log and 1.76 log, respectively. The combined strategy significantly reduced fibrinogen adsorption by up to 40% and platelet adhesion by 98%, thereby enhancing anti-fouling efficacy. The SNAP-impregnated samples infused with eugenol-NE demonstrated promising results and potential for biomedical applications.

## Abstract 2

This work introduces a multifunctional PDMS coating that uses a nanoemulsion platform to co-deliver nitric oxide and eugenol, enabling simultaneous reduction of bacterial adhesion and anti-fouling capabilities.

## Graphical abstract

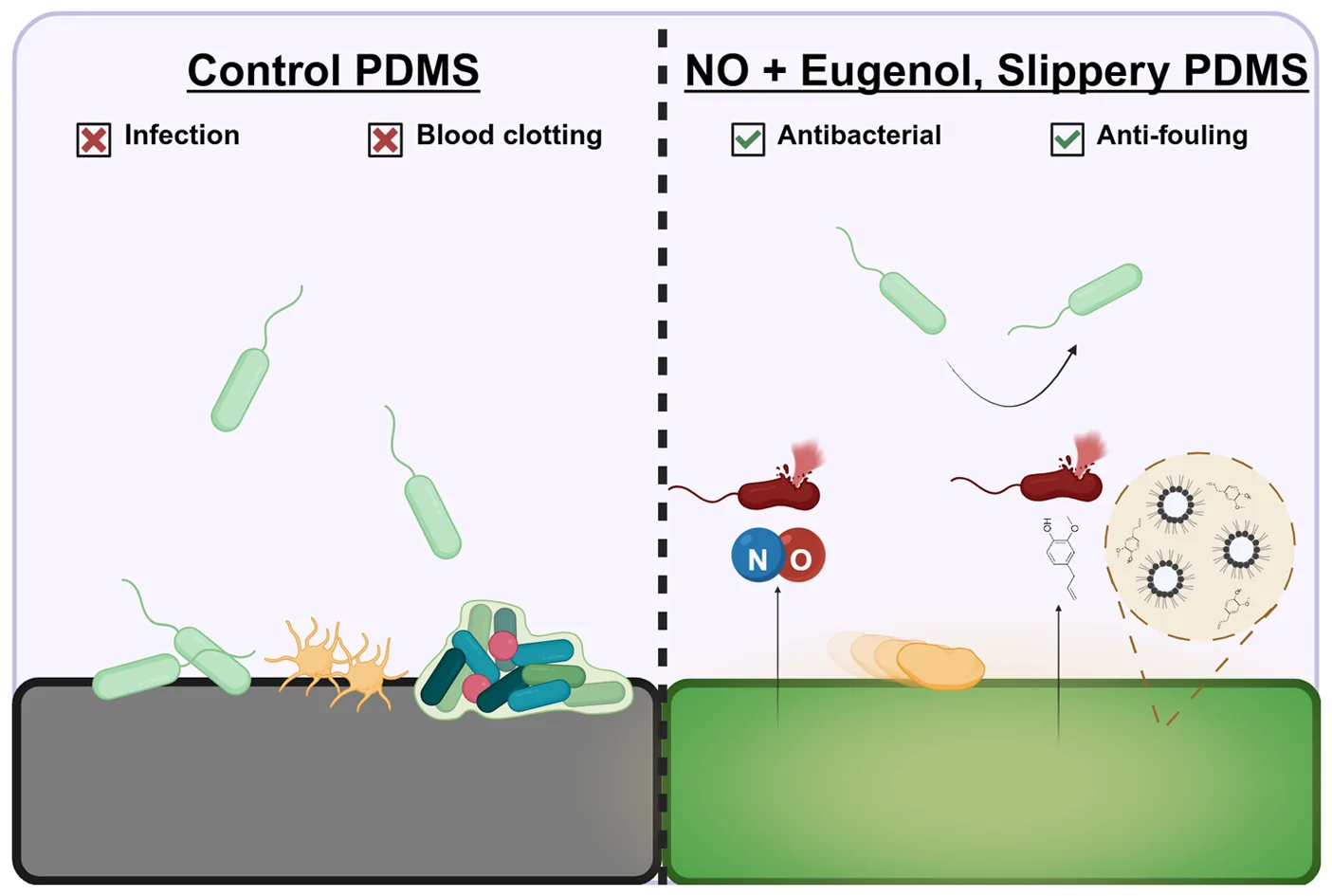

## 1. Introduction

Clinical infection and thrombosis remain some of the most common causes for prolonged hospitalization and increased treatment costs among patients, especially those with indwelling or implanted medical devices. According to the Centers for Disease Control and Prevention (CDC), hospital-acquired infections (HAIs), which are the onset of infection after admission to a hospital for treatment due to non-aseptic techniques, unsterile devices, or the body's natural microflora, affect approximately 2 million patients in the United States annually.^1^ About 50–70% of those reports are due to indwelling or implanted medical devices.^1^ If left untreated, the infections could worsen with the maturation of the bacteria to form biofilms protected by an extracellular polymeric substance (EPS) on the surface of the devices.^2^ The most accepted current treatment for these infections in healthcare settings is antibiotic administration.^3^ While the antibiotic may be effective at killing the bacteria for a short time, bacterial mutations may occur to aid in developing antibiotic resistance.^4^ If a biofilm forms, the large and bulky antibiotic molecule cannot penetrate through the EPS to deliver the optimized bactericidal dose, requiring over one thousand times the minimum inhibitory concentration (MIC).^3,5^ Despite these concerns, professionals still prescribe antibiotics as the standard treatment.

Another critical issue for blood-contacting devices is thrombosis, the aggregation of blood cells, plasma proteins, and platelets to form a clot.^6,7^ Approximately 2 million Americans experience thrombosis annually, with up to 300 000 of the cases leading to mortality.^8^ These clots can lead to embolism, device occlusion, and ultimately device failure.^9^ Prophylactic measures to prevent thrombosis or treatment of thrombus formation is typically the systemic administration of anticoagulants, such as heparin, warfarin, or argatroban.^10–12^ However, with systemic administration of these drugs comes the adverse effects of excessive bleeding, bruising, or unintentional platelet consumption.^13,14^

Over the last decade, new strategies to combat infection and thrombosis without relying on antibiotics or anticoagulants have been explored. One such strategy is the facile incorporation of nitric oxide (NO) in the form of donor molecules into medically relevant polymers, such as silicone rubber or polyurethane.^15–20^ As an endogenous free radical gasotransmitter, NO is known for its short half-life and inherent antibacterial and platelet-modulating properties to demonstrate excellent anti-infection potential and reduction of thrombotic events in biomaterials.^15–20^ A class of NO donors called *S*-nitrosothiols (RSNO), including *S*-nitroso-*N*-acetylpenicillamine (SNAP), has been widely researched and shows promising *in vitro* and *in vivo* efficacy.^21–23^ Since NO is incorporated into polymers *via* NO donor molecules, its release can be tuned to mimic physiological levels produced endogenously by the endothelium.^24^

While NO offers antibacterial and thrombus-reducing efficacy in the form of a gaseous molecule, other liquid antibacterial and anti-platelet molecules from natural compounds have also been explored for their antibacterial and platelet-modulating properties. One of these compounds is eugenol, which is the most abundant compound extracted from clove oil.^25,26^ Eugenol is a natural, aromatic phenolic compound^25^ that is highly soluble in organic solvents^27^ and has been widely used as an antimicrobial and antiseptic.^26,28^ Its antimicrobial efficacy is highly potent, with previous literature reporting MIC values of 0.25 μg mL^−1^ against *Staphylococcus aureus* (*S. aureus*) and 0.125 μg mL^−1^ against *Escherichia coli* (*E. coli*).^29^ In addition to the antimicrobial properties, eugenol exhibits some platelet-modulating properties to inhibit platelet activation through the suppression of key signaling pathways.^30,31^

While both NO-releasing materials and eugenol have excellent antibacterial and platelet-modulating properties, neither the NO molecule nor eugenol have anti-fouling capabilities to combat general biofouling. Therefore, anti-fouling techniques, such as liquid infusion for the fabrication of slippery surfaces, must be employed in combination with these technologies to comprehensively address the clinical challenges. Inspired by the *Nepenthes* pitcher plant, liquid infusion of a lubricant into a polymeric substrate involves swelling a lubricant oil within the polymer matrix to create a lubricant reservoir.^32^ The lubricant then creates a barrier layer at the surface and prevents many liquids and foulants from adhering.^16,32^ While most liquid-infused slippery surfaces only use one lubricant, such as silicone oil or perfluorodecalin,^16,32,33^ recent reports explore the use of a water-in-oil (w/o) nanoemulsion (NE) as the swelling medium.^34–36^ The w/o NE uses a lubricant oil (*e.g.*, *n*-hexadecane) as the bulk phase while nano-sized aqueous droplets, with the ability to load water-soluble bioactive agents, are dispersed throughout the continuous oil. These NEs have been incorporated into several polymer substrates, including silicone rubber,^35^ polytetrafluoroethylene (PTFE) membranes,^34^ and expanded PTFE^36^, and have shown comparable antibacterial and anti-fouling capabilities.

Although NO-releasing polymers and liquid-infused slippery surfaces have each independently demonstrated reductions in early-stage thrombogenic events and infection, prior platforms have largely relied on inert lubricants or a single bioactive strategy. Recent nanoemulsion-infused systems have enabled the incorporation of hydrophilic bioactives; however, the integration of hydrophobic antimicrobial agents within the lubricant phase, particularly in combination with NO-releasing substrates, remains largely unexplored. Unlike direct incorporation of hydrophobic agents such as eugenol into PDMS, the nanoemulsion facilitates more uniform distribution and can contribute to surface slipperiness, which is critical for reducing both bacterial and platelet adhesion. Moreover, few reports have demonstrated a multifunctional system that simultaneously delivers antimicrobial and anti-platelet activity while preserving durable anti-fouling behavior. Therefore, there is a need for platform techniques that integrate both active and passive mechanisms without increasing fabrication complexity or compromising biocompatibility.

**Fig. 1:**
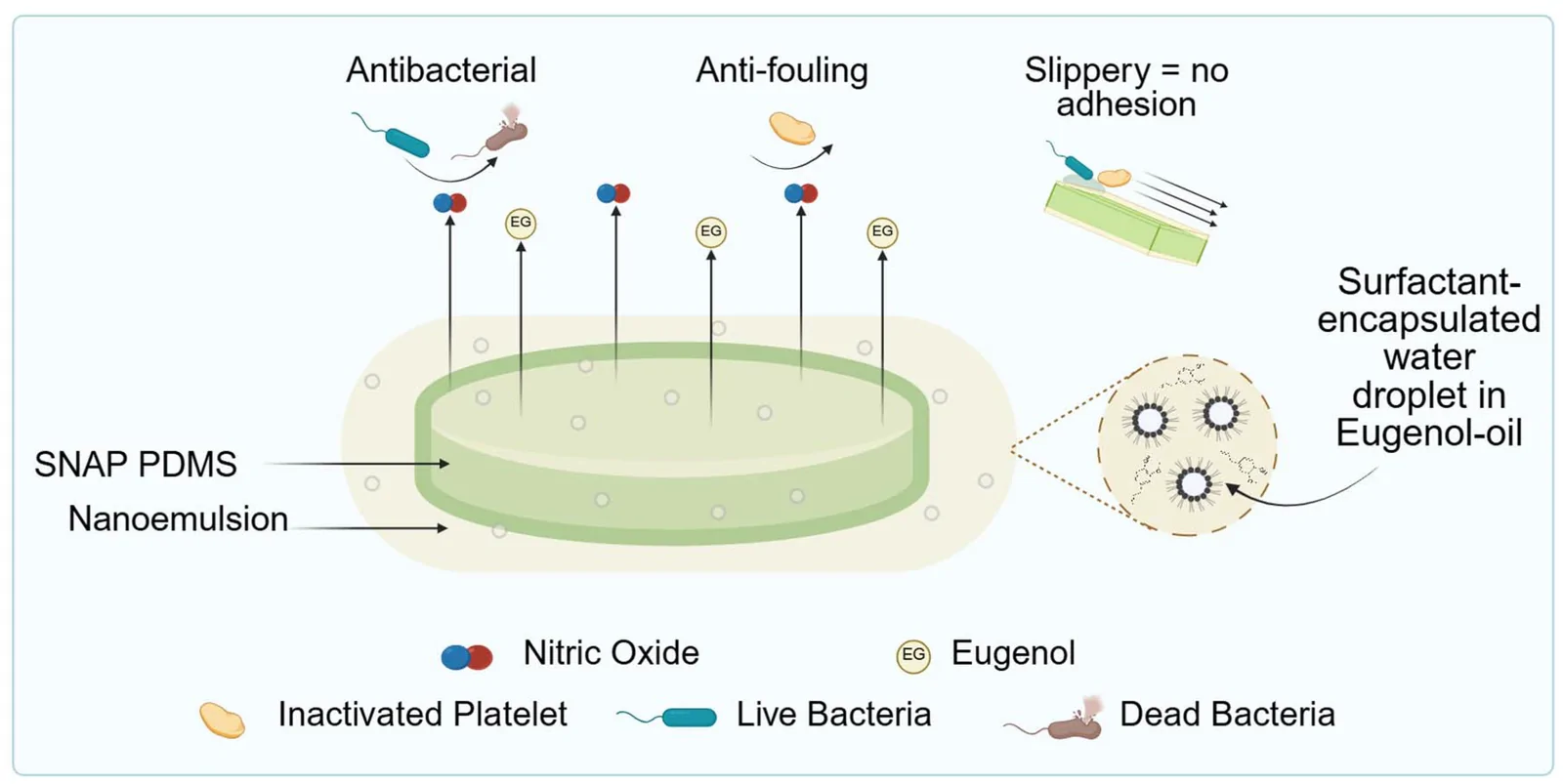
Proposed material design incorporating bioactive eugenol into an *n*-hexadecane-based nanoemulsion and swelled into an NO-releasing substrate.

In this work, the bioactive compound eugenol is incorporated into the lubricant phase of an *n*-hexadecane-based NE and infused into a SNAP-impregnated silicone rubber (Fig. 1). This combination of bioactive eugenol and NO release, along with passive slippery behavior from the NE infusion, was used to fabricate a multifunctional substrate capable of enhanced antibacterial efficacy, improved platelet modulation, and reduced surface fouling. Here, eugenol was dissolved in *n*-hexadecane and used as the continuous phase of the NE. The NE was fabricated by slowly incorporating the oil phase with the aqueous phase under low-shear magnetic stirring. Following magnetic stirring, the NE was sonicated using a high-energy probe sonicator to disperse any microdroplets. The eugenol-NE was then swelled into the SNAP-impregnated silicone rubber, and its surface properties were characterized by sliding angle over 24 h. The NO release was measured over 24 h using a Nitric Oxide Analyzer (NOA), and the antibacterial efficacy was assessed by a 24 h bacterial adhesion assay against the Gram-positive *S. aureus* and the Gram-negative *E. coli*. The biocompatibility of the substrate was evaluated using L-929 mouse fibroblast cells in a 24 h leachate exposure assay and porcine platelet-rich plasma in a plasma-free calcein assay. It is expected that the combination of eugenol, NO, and slippery behavior will prevent bacterial viability while maintaining biocompatibility in the aforementioned studies.

## 2. Materials & methods

### 2.1. Materials

Dimethyl sulfoxide (DMSO, 99.9%), thiazolyl blue tetrazolium bromide 98%, *n*-hexadecane (99%), sodium nitrite (≥97%), hydrochloric acid (37%), sulfuric acid, polyoxyethylene (20) sorbitan monooleate (Tween 80), sorbitan monooleate (Span 80), fibrinogen from human plasma (>90%), sodium citrate dihydrate, and Luria Bertani (LB) broth media and agar were purchased from Sigma Aldrich (St Louis, MO). Phosphate-buffered saline (PBS) 10 mM with 100 μM ethylenediaminetetraacetic acid (EDTA) was used for all material characterization experiments and NO analyzer studies; EDTA was included to chelate metal ions and minimize unintended catalytic decomposition of SNAP, ensuring more controlled NO release measurements. *N*-Acetylpenicillamine was obtained from Pharmablock (West Chester, PA). Calcium and magnesium-free PBS (CMF-PBS, 1×), calcein-AM, and Hank's Balanced Salt Solution (HBSS, calcium and magnesium-free) were purchased from Corning Incorporated (Manassas, VA). Eugenol terpene isolate was obtained from Floraplex Terpenes. Tygon 3350 Silicone Tubing was purchased from Saint-Gobain (Malvern, PA). Eagle's Minimum Essential Medium (EMEM), fetal bovine serum (FBS), and penicillin–streptomycin (P/S) were purchased from VWR (Radnor, PA). NCTC clone 929 mouse fibroblast cells (L-929, ATCC CCL-1), bacterial strain *Escherichia coli* (*E. coli*, ATCC 25922), and *Staphylococcus aureus* (*S. aureus*, ATCC 6538) were obtained from American Type Culture Collection (ATCC, Manassas, VA). Fresh porcine whole blood was collected from the University of Georgia Swine Unit (Athens, GA).

### 2.2. Preparation of sample materials

#### 2.2.1. Fabrication of Sylgard 184 PDMS

To prepare the substrate used for all studies, the DOW Chemical Sylgard 184 PDMS, herein referred to as PDMS, was fabricated. Briefly, the elastomer base was combined with the curing agent in a 10 : 1 ratio. After thoroughly mixing the components, the mixture was cast into a glass mold and cured at 100 °C for 1 h. The thickness of the PDMS films were measured with a micrometer (Table S2), and the samples were stored at room temperature.

#### 2.2.2. *S*-nitroso-*N*-acetylpenicillamine (SNAP) synthesis

The NO donor *S*-nitroso-*N*-acetylpenicillamine was synthesized using a previously reported protocol^37^ with modifications. Briefly, 5 g of the precursor *N*-acetylpenicillamine (NAP) was dissolved in 60 mL of methanol. With the dissolved NAP in an ice bath, 5 mL of H_2_SO_4_ and 20 mL of HCl were added dropwise to the solution. Then, 5 g NaNO_2_ dissolved in 40 mL DI water was slowly added to the dissolved NAP for nitrosation. The solution was reacted for 8 h under gentle nitrogen gas agitation to promote methanol evaporation and protected from light. After the reaction period, the resulting SNAP crystal was vacuum-filtered, washed with chilled DI water, desiccated, and stored at −20 °C, protected from light.

The purity of the SNAP was verified by area-under-the-curve analysis. Approximately 3.5 mg of SNAP was dissolved in 10 mM PBS w/100 μM EDTA. A solution containing 50 mM CuCl_2_ and 10 mM cysteine was prepared in DI water. In the NOA sample chamber, 10 mM PBS w/o EDTA was added alongside the CuCl_2_ and cysteine solutions. The SNAP solution was injected in the NOA sample chamber (25 μL), was allowed to release NO, and come back down to baseline. This was repeated 3 more times to confirm NO release. The area under each peak was taken to calculate the number of moles of NO and compared to the theoretical number of moles of NO (Fig. S1).

#### 2.2.3. SNAP impregnation into PDMS

To incorporate the NO-donor SNAP into the PDMS, a simple impregnation method was used following prior methods.^38^ First, a 50 mg mL^−1^ SNAP solution in tetrahydrofuran (THF) was prepared. The PDMS samples were added to the SNAP solution, protected from light, and allowed to be impregnated by the SNAP solution under gentle rocking for 24 h. The samples were then removed from the SNAP–THF solution, and the solvent was allowed to evaporate for 24 h. Finally, the resulting SNAP-impregnated samples were rinsed in methanol and DI water to remove any SNAP surface crystals. The samples were desiccated to remove residual solvent and stored at −20 °C.

#### 2.2.4. Nanoemulsion (NE) fabrication

The NE was fabricated following a reported protocol^34^ with modifications. Briefly, polyoxyethylene (20) sorbitan monooleate (Tween 80; 7.5 parts by weight) and sorbitan monooleate (Span 80; 22.5 parts by weight) were dissolved in *n*-hexadecane or a *n*-hexadecane and eugenol mixture (2% v/v eugenol, 70 parts by weight total) and vortexed to thoroughly mix. The surfactant mixture in the *n*-hexadecane was then vacuum-filtered through a 0.1 μm PTFE filter. Then, 10 mM PBS with 100 μM EDTA (5% v/v) was added to the vial, followed by the *n*-hexadecane/surfactant mixture at a rate of 200 μL per 20 s, with continuous magnetic stirring at 500 rpm. The emulsion was then sonicated using a probe sonicator (Split Type Ultrasonic Processor, Vevor, Shanghai, China, 50%, 19.5 kHz) for 2 min. The nanoemulsion was then stirred at 500 rpm for 1 h to stabilize the nanodroplets. The unloaded NE and the eugenol-loaded NE are herein referred to as NE and 2%EG, respectively.

### 2.3. Nanoemulsion characterization

#### 2.3.1. Dynamic light scattering (DLS)

Dynamic light scattering, based on the Brownian motion of dispersed particles, was used to determine the hydrodynamic diameter of the nanodroplets in the nanoemulsions. As particle movement increases, the resulting fluctuations in scattered light intensity also increases, allowing particle size to be inferred.^39^ The prepared NEs were diluted with *n*-hexadecane in a 1 : 200 ratio, and the diluted samples were analyzed using a Zetasizer Nano ZS (Malvern Panalytical, Malvern, Worcestershire, United Kingdom). Three consecutive measurements were performed to verify nanoemulsion formation, typically defined by hydrodynamic diameters below 200 nm. All data was processed using the Zetasizer software.

#### 2.3.2. Fourier transform infrared (FTIR) spectroscopy

Fourier transform infrared spectroscopy (FTIR) was performed using a Spectrum 3 spectrometer (PerkinElmer, Waltham, MA) equipped with a universal attenuated total reflectance (UATR) accessory to verify the presence of the nanoemulsion within the polymer matrix. UATR-FTIR was also used to characterize the nanoemulsion components, *n*-hexadecane, Tween 80, Span 80, and eugenol, for comparison with the polymer composites. Spectra were collected over a range of 4000–650 cm^−1^ at a resolution of 4 cm^−1^, with 32 scans acquired for each sample. All spectral data were processed using the PerkinElmer Spectrum 10 software.

### 2.4. PDMS-oil impregnation

To determine the total time required to fully impregnate the PDMS substrates with the NEs, the swelling ratios and rates were calculated. The weights of fresh samples were measured and recorded, and the samples were incubated in the NEs. The weight of the samples was measured every 2 h for 24 h. The swelling ratios and rates were calculated according to eqn (1) and (2), where *m*_*x*_ is the sample weight at a given time point, *m*_initial_ is the sample mass prior to incubation in the NEs, ΔSR is the change in swelling ratio, and Δ*t* is the change in time. The controls and test groups are herein referred to as PDMS, SNAP, PDMS-NE, SNAP-NE, PDMS-2%EG, and SNAP-2%EG (Table S1).1
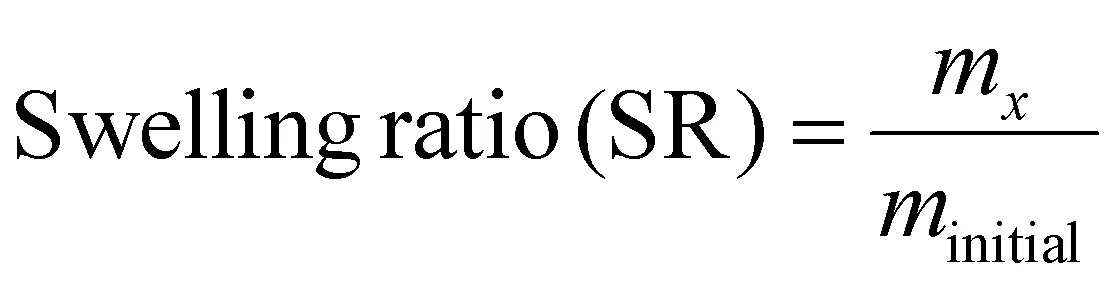
2
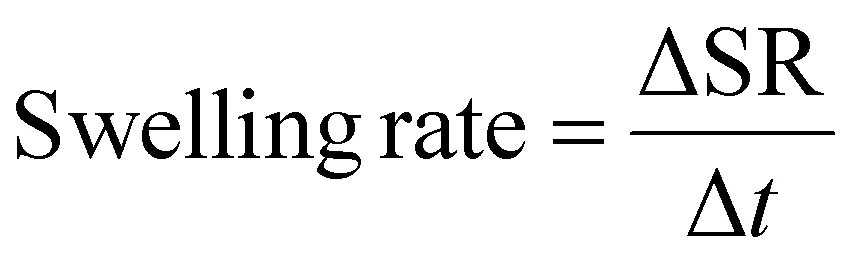


### 2.5. Cryogenic scanning electron microscopy

Confirmation of the nanoemulsion stability after swelling was confirmed through Cryo-SEM using a Hitachi SU3900 (Hitachi High-Tech America, Inc., Schaumburg, Illinois, U.S.A.). Samples were mounted onto aluminum stubs using a mixture of O.C.T compound and colloidal graphite to ensure stability during the freezing and imaging processes. Samples were then sublimated for 4 minutes at −90 °C, followed by sputter coating with platinum for 120 s at 10 mA. Imaging was performed at an accelerating voltage of 20 kV and a working distance of 13–15 mm.

### 2.6. Static water contact angle

The static water contact angle measurements of the substrates were measured using a Contact Angle Goniometer (Ossila Ltd, Sheffield, England). Briefly, samples were mounted on the goniometer stage, and the camera was focused. A 5 μL droplet of DI water was pipetted on the surface, and a video was recorded and analyzed to determine the water contact angle. This was repeated for all technical replicates.

### 2.7. Sliding angle

While static water contact angles provide some insight into a material's surface wettability, the sliding angle of a slippery surface characterizes its slippery behavior. All sample groups were measured by placing the sample on a digital protractor with a 5 μL droplet of methylene blue-dyed DI water on the surface. The digital protractor was increased in 1° increments until the water droplet slid on the surface. The angle at which the droplet began to slide was recorded as the sliding angle. For all NE-swelled samples, the NE was removed, and the samples were gently dried to remove excess lubricant. The sliding angle was then measured as described. To determine the slippery behavior stability of the NE-swelled samples, the sliding angle was measured again after 24 h of incubation in 10 mM PBS with 100 μM EDTA at 37 °C. The samples were removed from the PBS, gently dried to remove excess PBS, and measured.

To evaluate the proposed concept's slippery behavior on a curved surface, Tygon 3350 tubing was prepared. The tubing was impregnated with SNAP and swelled with a eugenol-loaded nanoemulsion, and all controls were also prepared. The sliding angle was then measured from the surface of the modified tubing in a similar manner.

### 2.8. SNAP loading

The total amount of SNAP impregnated into the PDMS films was assessed to determine whether swelling induced by the NEs affected the NO donor reservoir. Briefly, the NO-releasing samples (SNAP, SNAP-NE, and SNAP-2%EG) were incubated in THF for 2 h. The absorbance of the leachates was measured on an Agilent Cary 60 UV-vis spectrometer (Agilent Technologies, Santa Clara, CA) at 340 nm. The absorbance was converted to a SNAP concentration *via* Beer–Lambert's law, and the amount of SNAP that swelled into the PDMS was calculated.

### 2.9. SNAP leaching

As the SNAP molecule is not covalently bound to the polymer matrix, it can be prone to diffusion from the bulk material; therefore, a leaching study was conducted. Briefly, SNAP-containing samples (SNAP, SNAP-NE, and SNAP-2%EG) were incubated in 10 mM PBS with 100 μM EDTA (2 mL), herein referred to as leachates, for set time points. At each time point, the absorbance of the leachates was measured using an Agilent Cary 60 UV-vis spectrometer (Agilent Technologies, Santa Clara, CA) at 340 nm, and the cumulative SNAP leached was calculated over 24 h.

### 2.10. Eugenol loading

The total amount of eugenol in the PDMS films was assessed. Briefly, the 2%EG-swelled samples (PDMS-2%EG, SNAP-2%EG) were incubated in THF for 2 h. The absorbance of the leachates was measured on an Agilent Cary 60 UV-vis spectrometer (Agilent Technologies, Santa Clara, CA) at 280 nm. The absorbance was converted to a concentration of eugenol using Beer–Lambert's law (molar absorptivity in THF = 286.76 M^−1^ cm^−1^), and the amount of eugenol swollen into the PDMS was calculated. The absorbance of SNAP at 280 nm was subtracted from the total absorbance in the SNAP-2%EG samples to ensure that the contribution from SNAP was taken into account.

### 2.11. Eugenol remaining

The amount of eugenol remaining within the 2%EG-swelled samples (PDMS-2%EG, SNAP-2%EG) after incubation in physiological conditions (10 mM PBS w/100 μM EDTA @ 37 °C) for 24 h was quantified. Briefly, the PDMS-2%EG and SNAP-2%EG samples were incubated in 10 mM PBS w/100 μM EDTA @ 37 °C for 24 h, protected from light. After incubation, the samples were removed from the PBS, transferred to THF, and incubated for 2 h to extract the remaining eugenol from the substrates. The absorbance of the extracts was measured on an Agilent Cary 60 UV-vis spectrometer (Agilent Technologies, Santa Clara, CA) at 280 nm. The absorbance was converted to a eugenol concentration using the same procedure as above.

### 2.12. Nitric oxide (NO) detection

The NO release from SNAP, SNAP-NE, and SNAP-2%EG samples was measured using the gold-standard Zysense Nitric Oxide Analyzer (NOA) 280i (Frederick, CO) *via* the chemiluminescence detection method. The NO-releasing samples were initially submerged in 10 mM PBS with 100 μM EDTA at 37 °C. A nitrogen bubbler and sweep gas were introduced into the solution at a flow rate of 200 mL min^−1^ to displace any NO dissolved in the liquid phase and direct it into the NOA. The NO release was monitored until a steady release rate was achieved. After measurement, the samples were kept in 10 mM PBS with 100 μM EDTA at 37 °C, protected from light between time points. The NO release was subsequently quantified 24 h after the initial measurement.

### 2.13. Eugenol loading after UV sterilization

To evaluate the potential for UV sterilization to affect the eugenol payload, a similar loading experiment was conducted. Briefly, 2%EG-swelled samples were UV sterilized for 30 min (15 min on each side). After sterilization, the samples were incubated in THF for 2 h to extract the eugenol that swelled into the substrates. The absorbance of the extracts was then measured and quantified on an Agilent Cary 60 UV-vis spectrometer (Agilent Technologies, Santa Clara, CA) at 280 nm. The absorbance was converted to a eugenol concentration using the same procedure as above.

### 2.14. NO release after UV sterilization

Similarly, the NO release after UV sterilization was assessed for all SNAP-containing samples. Essentially, the SNAP-containing samples were UV sterilized for 30 min (15 min on each side). After sterilization, the NO released from each sample was measured using the gold-standard Zysense Nitric Oxide Analyzer (NOA) 280i (Frederick, CO) *via* chemiluminescence detection. The conditions for this study were similar to those of the procedure above.

### 2.15. *In vitro* cytotoxicity evaluation

Cell cytocompatibility of the SNAP-2%EG samples was evaluated using NCTC clone 929 mouse areolar fibroblast cells (L-929) in accordance with ISO 10993-5 guidelines.^40^ The L-929 cells were thawed and cultured in EMEM supplemented with 10% fetal bovine serum and 1% penicillin–streptomycin (complete EMEM). Cells were seeded in 96-well plates at a density of 1 × 10^4^ cells per well and incubated for 24 h at 37 °C with 5% CO_2_.

The samples (PDMS, SNAP, PDMS-NE, SNAP-NE, PDMS-2%EG, and SNAP-2%EG) were sterilized with 70% ethanol, then exposed to UV light for 30 min (15 min per side). Each sample was then incubated in complete EMEM (1 mL) for 24 h at 37 °C, 5% CO_2_. The cells were then exposed to the collected leachates for an additional 24 h under the same conditions.

Cell metabolic activity following leachate exposure was quantified using the MTT colorimetric assay. After removing the leachates, 100 μL of MTT working solution (0.5 mg mL^−1^) was added to each well and incubated for 2.5 h at 37 °C, 5% CO_2_. After incubation, the MTT solution was removed and replaced with 100 μL DMSO to dissolve the resulting formazan crystals, and the plate was agitated on an orbital shaker for 10 min to ensure complete dissolution. The absorbance of the plate was measured at 570 nm with a 690 nm reference using a BioTek Cytation 5 Imaging Reader (Winooski, VT). Relative cell viability was calculated using eqn (3), where ABS_sample_ is the absorbance of the cells exposed to test sample leachates, ABS_control_ is the absorbance of untreated control cells, and ABS_blank_ is the absorbance of wells containing only DMSO. Final data are reported as the mean ± standard deviation (*n* ≥ 4 samples).3
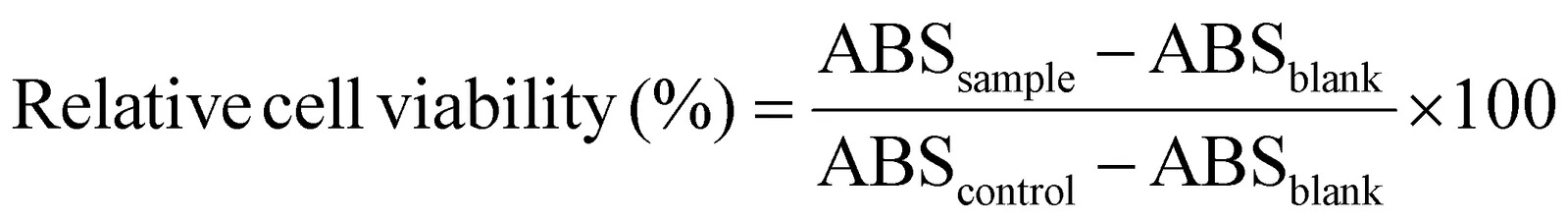


### 2.16. *In vitro* hemocompatibility assessment

#### 2.16.1. Fibrinogen (Fg) adsorption

To assess the prevention of fibrinogen adsorption on SNAP-2%EG and its controls, a fluorescent fibrinogen adsorption assay (FITC-Fg) was performed. Briefly, all test samples were incubated at 37 °C in CMF-PBS for 1 h to reach surface saturation. A working concentration (2 mg mL^−1^) of Fg was prepared consisting of 1 : 10 FITC-labeled Fg to unlabeled Fg. A standard curve of the FITC-Fg was prepared with the working solution and serially diluted in a 1 : 1 ratio with CMF-PBS, plated in triplicate, and incubated at 37 °C for 90 min. The samples were also incubated in the working solution (200 μL) at 37 °C for 90 min. After incubation, the non-adsorbed Fg was gently removed with several washes with CMF-PBS. The fluorescence intensity of the samples was then measured using a BioTek Cytation 5 Imaging Reader (Winooski, VT) with excitation at 490/20 and emission at 525/20. The amount of Fg adsorbed was quantified and normalized to the surface area. Final data are reported as the mean ± standard deviation (*n* = 8 samples).

#### 2.16.2. Platelet adhesion

The prevention of platelet adhesion of SNAP-2%EG and its controls was evaluated using the calcein assay, a plasma-free, fluorescent method according to ISO 10993-4 standards.^41^ In short, the control and test samples were first incubated in an unlabeled Fg solution (3 mg mL^−1^) for 90 min. Non-adsorbed Fg was removed with HBSS, and the fluorescence of the samples was measured using a BioTek Cytation 5 Imaging Reader (Winooski, VT) in a black 96-well plate to get a background (gain = auto, Ex = 485, Em = 535).

All procedures involving porcine whole blood and its derivatives were approved by the University of Georgia Institutional Animal Care and Use Committee (IACUC approval #A2023 11-012-A7). Porcine whole blood was collected and citrated in a 1 : 9 ratio (3.2% sodium citrate) to prevent premature coagulation. The blood was then centrifuged to separate the red blood cells, the buffy layer, and the plasma. The platelet-rich plasma (PRP) was collected and combined with HBSS; the PRP was then centrifuged again. Simultaneously, the calcein-AM stock was prepared. Cell-culture grade DMSO (20 μL) was added to calcein-AM (50 μg). The calcein-AM dilution in HBSS was then prepared to a final concentration of 5 μM. After centrifugation, the platelet-poor plasma (PPP) was removed, and the resulting platelet pellet was resuspended in the HBSS/calcein solution and incubated for 30 min (37 °C, protected from light).

After incubation, the supernatant containing the plasma-free platelets was transferred to a new tube, leaving behind the insoluble portion. The platelets were then centrifuged once more. The supernatant containing the excess HBSS/calcein solution was removed, and the now calcein-tagged platelets were resuspended in HBSS. The platelets were diluted to a working concentration of 1 × 10^8^ platelets mL^–1^ and exposed to the samples. Simultaneously, a standard curve was prepared by serially diluting the working solution in a 1 : 1 ratio of HBSS and plated in triplicate. The samples were incubated for 90 min at 37 °C, protected from light; after incubation, the non-adherent platelets were removed, and the fluorescence of the samples was measured using the BioTek Cytation 5 Imaging Reader (Winooski, VT, gain = auto, Ex = 485, Em = 535). The number of platelets adhered was quantified and normalized to the surface area. Final data are reported as the mean ± standard deviation (*n* = 8 samples).

### 2.17. *In vitro* bacterial adhesion assessment

The antibacterial performance of the SNAP-2%EG samples and controls were assessed against *Staphylococcus aureus* (ATCC 6538) and *Escherichia coli* (ATCC 25922) using a 24 h bacterial adhesion assay. To prepare the inoculum, a single colony from an agar plate was transferred into LB broth and grown to mid-log phase. Culture density was verified by measuring absorbance at 600 nm with a UV-vis spectrophotometer (Thermo Scientific Genesys 10S UV-vis). Cells were then washed with PBS and adjusted to an optical density of 0.1 before exposing them to the polymer samples.

The PDMS, SNAP, PDMS-NE, SNAP-NE, PDMS-2%EG, and SNAP-2%EG were anchored with sterile needles to prevent floating, immersed in the bacterial suspensions, and incubated for 24 h at 37 °C with shaking at 150 rpm. After incubation, the samples were rinsed to remove non-adherent bacteria and transferred to sterile PBS. Adherent bacteria were detached using an Omni-TH homogenizer (Omni, Kennesaw, GA) for 1 min, followed by vortexing for an additional 1 min. The recovered bacterial suspensions were plated on LB agar using a spiral plater (Eddy Jet 2 W, IUL Instruments) and incubated overnight at 37 °C. Colony-forming units (CFUs) were counted the following day, and the bacterial adhesion was expressed as CFU cm^–2^ using eqn (4), where CFU_control_ is the adhesion on untreated PDMS and CFU_sample_ is the adhesion on the tested materials. Final data are reported as the mean ± standard deviation (*n* = 4 samples).

Tygon 3350 tubing was also prepared to evaluate its antibacterial efficacy. The control PDMS, SNAP, PDMS-NE, SNAP-NE, PDMS-2%EG, and SNAP-2%EG tubing samples were prepared as previously described. Similar exposure to *S. aureus* solutions was performed, and the bacteria that adhered to the tubing were quantified. The adhesion was expressed as CFU cm^–2^, as well.4
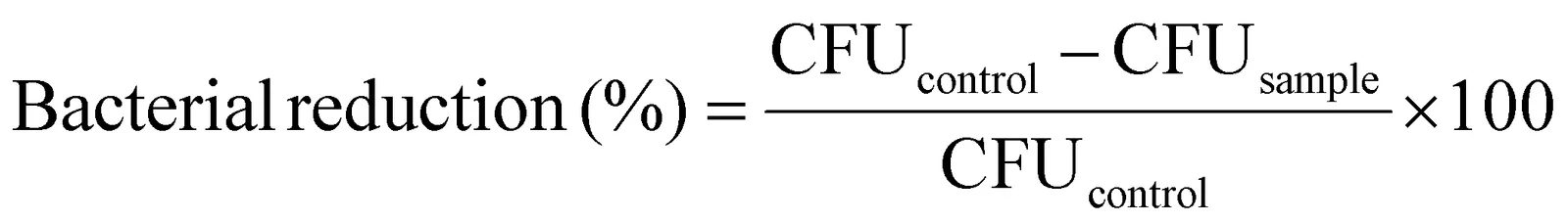


### 2.18. Statistical analysis

All experiments performed in this study are presented in *n* ≥ 3, and all statistical analyses were performed using GraphPad Prism 9 (GraphPad Software, San Diego, CA) to determine statistical significance using a one-way ANOVA test. Data are presented as mean ± standard deviation. A *p*-value of <0.05 is considered significant for all data presented in this study.

## 3. Results & discussions

### 3.1. Fabrication and characterization of the eugenol-NE swelled PDMS

As NEs have the physical limitation of a small aqueous phase, a water-soluble bioactive agent would have limited efficacy. In this case, only 5% v/v of the total NE is the aqueous phase; therefore, there is a need for enhanced bioactivity within these NE platforms. Since eugenol is readily soluble in organic solvents and has potent antimicrobial potential, it was chosen as a bioactive lubricant to be incorporated into the *n*-hexadecane-based NE.

Upon fabrication of the NEs, no visible aggregates of the aqueous micelles were observed. However, the hydrodynamic diameter of the aqueous droplets was assessed using Dynamic Light Scattering (DLS), which is governed by Brownian motion.^39^ With increased random motion of the particles, or in this case, the nanodroplets, an increase in light scattering is observed, tracked, and correlated to the nanodroplets’ size.^39^ Nanoemulsions have droplet sizes in the range of 20–200 nm that are typically stabilized with surfactants that create micelles around the droplet.^42,43^ Dynamic light scattering measurements confirmed the formation of nanoemulsions with hydrodynamic diameters of 87.11 ± 3.05 nm and 137.83 ± 6.46 nm for the plain NE (NE) and the eugenol-incorporated NE, respectively (2%EG, Fig. S2). The average polydispersity index (PDI), which correlates to sample uniformity, of the NE was 0.23 ± 0.03 while the PDI of the 2%EG was 0.39 ± 0.03 (Fig. S2). With these lower PDI values and more uniform nanodroplets, it is less likely that the NEs will become unstable and phase separate in a short time frame. It is hypothesized that the hydroxyl group from the eugenol was attracted to the hydrophilic aqueous droplet and aided in micelle formation with the Tween 80 and Span 80. Because of the bulkier structure of eugenol, steric hindrance might have contributed to the slightly larger droplet size seen in the 2%EG.

**Fig. 2:**
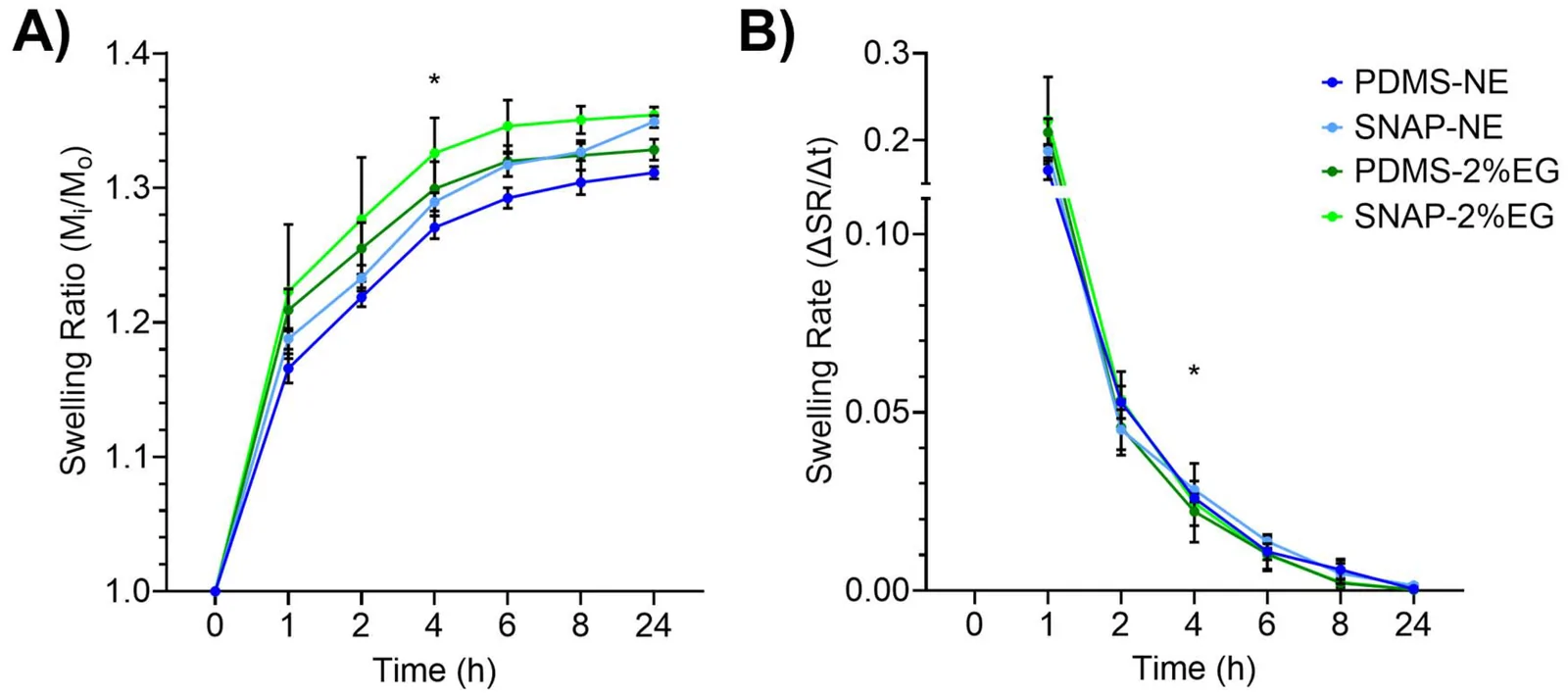
Swelling (A) ratio and (B) rate of the NE-swelled samples. Once the samples were immersed in the NEs, only 4 h were required to fully and sufficiently swell the silicone rubber-based films with the *n*-hexadecane-based NE. There was a significant change in the swelled ratio between *h*2 and *h*4, but the mass of the samples did not significant change after 4 h (*n* = 4, * *p* < 0.05 *vs*. time = 2 h).

Once the formation of the NEs was confirmed, they were then incorporated into the PDMS substrates. The nanoemulsion was visualized within the polymer matrix using cryo-SEM to determine the integrity of the droplets. It is hypothesized that the polymer network provided sufficient free volume to accommodate dispersed nanodroplets and may have limited large-scale coalescence (Fig. S3). Although typical liquid infusion into silicone rubber substrates uses silicone oil due to the similar chemical nature and properties,^16^ other lubricants, such as the aliphatic *n*-hexadecane, can also be swelled into PDMS despite the decreased chemical interactions. The *n*-hexadecane-based NEs could still swell into the PDMS and SNAP PDMS substrates through physical liquid infusion and capillary action. It was determined that 4 h was sufficient to fully swell the polymer substrates with the NEs (Fig. 2A and B). Compared to silicone oil, the swelling time is greatly reduced,^16^ which can help to preserve the bioactivity of the NO and eugenol. Although swelling occurs relatively quickly, and the NE reservoir is limited, it still provides enough lubricant to restore surface lubrication lost through agitation or physical removal.

### 3.2. Fourier transform infrared spectroscopy (FTIR)

**Fig. 3:**
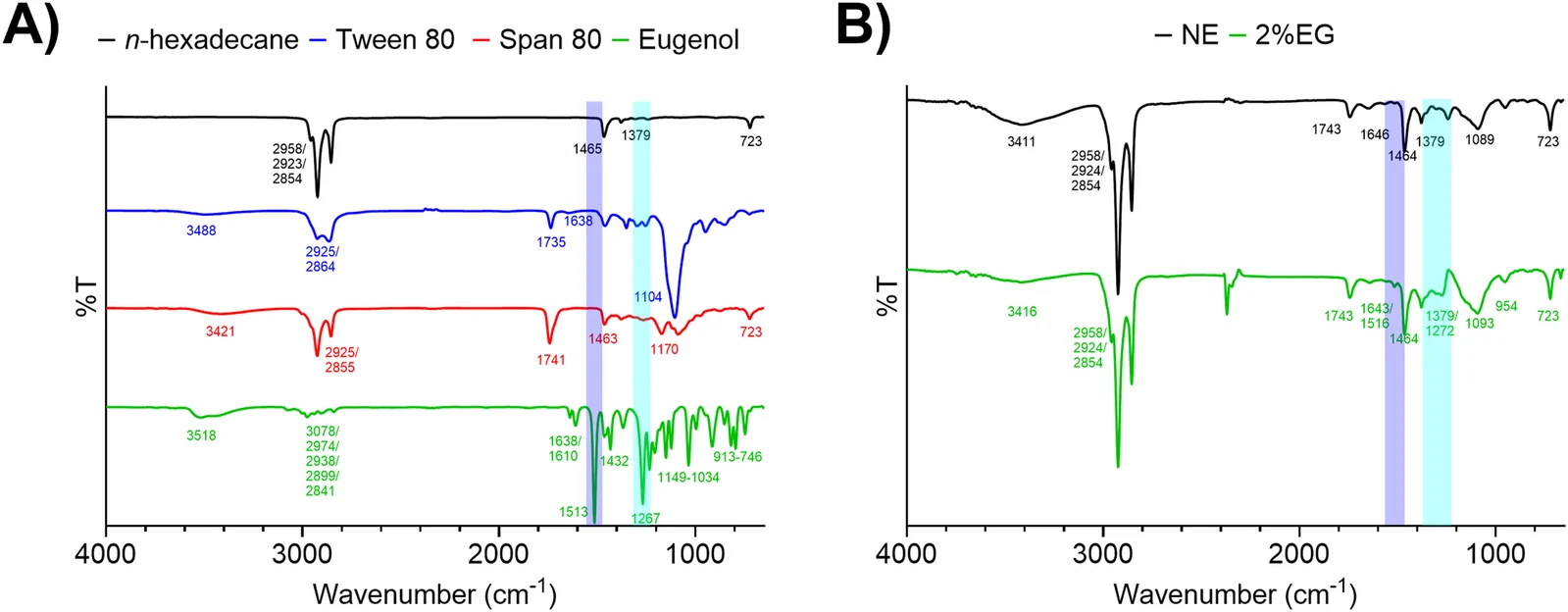
Fourier transform infrared spectra of the (A) NE constituents and (B) the NEs themselves. The NEs exhibited all of the characteristic peaks of *n*-hexadecane, Tween 80, and Span 80, and in the case of 2%EG, eugenol.

Universal attenuated total reflectance FTIR (UATR-FTIR) was used to study the chemical composition of the NEs. Both the plain NE and 2%EG showed the characteristic peaks associated with *n*-hexadecane, Tween 80, and Span 80 (Fig. 3A). A broad band from 3300–3400 cm^−1^ suggests the presence of O–H bonds, most likely due to the surfactants Tween 80 and Span 80. Several sharp peaks between 2850–3000 cm^−1^ are consistent with characteristic peaks of *n*-hexadecane, indicating the majority presence within the NEs, as well as several weaker peaks between 1300–1400 cm^−1^ (C–H and C–C stretching). Although present in a much smaller volume compared to *n*-hexadecane, characteristic peaks around 1512 cm^−1^ and 1270 cm^−1^ suggested the presence of eugenol in 2%EG, indicative of C–C double bond stretching and C–O stretching, respectively (Fig. 3B). These spectral changes suggest the successful incorporation of eugenol into the nanoemulsion.

### 3.3. Slippery behavior assessment

One of the challenges that indwelling medical devices face is surface fouling from biological media, such as blood. As a foreign material in the body, blood cells, platelets, protein, or bacteria can accumulate on the surface of the device, leading to clotting, infection, and ultimately device failure. While the wettability of a material could be indicative of the material's potential to accumulate biofoulants (Fig. S4), it does not give insight into the anti-fouling efficacy. Therefore, the slippery behavior of the SNAP-2%EG and the controls was assessed by measuring the sliding angle. The sliding angle is the angle at which a water droplet on a surface begins to slide as the incline increases. A sliding angle of 20° or less typically characterizes slippery behavior.^16^

**Fig. 4:**
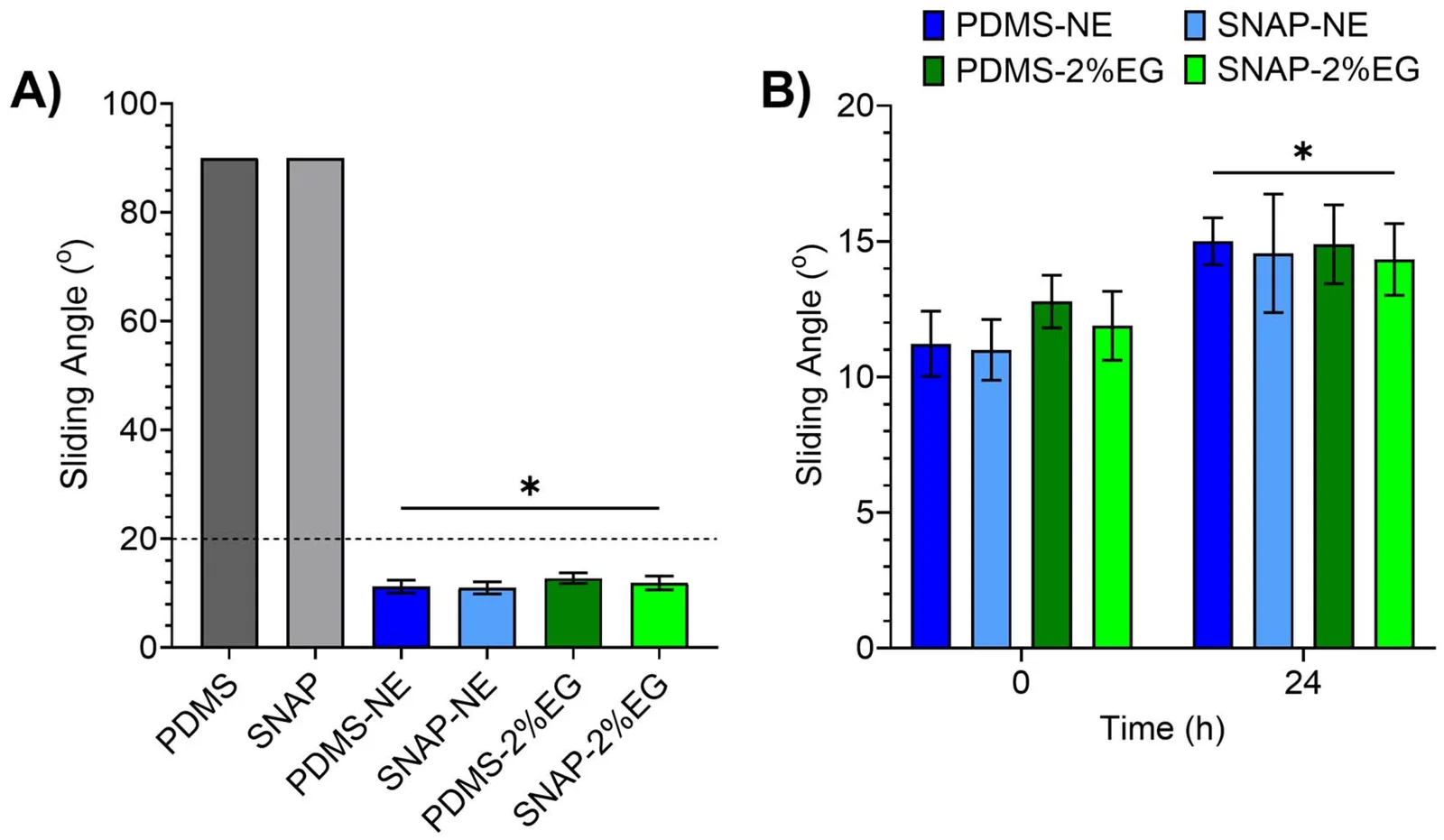
(A) Initial sliding angle of NE-swelled samples. While the control PDMS and SNAP did not exhibit any slippery behavior with sliding angles around 90°, all of the NE-swelled samples had low initial sliding angles below 20° (*n* = 9, * *p* < 0.05 *vs*. PDMS and SNAP). (B) Sliding angle stability of NE-swelled samples after incubation in physiological conditions. Although there was some lubricant depletion after incubation as seen with the slightly increased sliding angle, all sample types maintained slippery behavior with sliding angles below 20° (*n* = 9, * *p* < 0.05 *vs*. *t* = 0).

The PDMS and SNAP samples exhibited no slippery behavior, as shown by sliding angles of 90° (Fig. 4A). This means that the water droplet was completely pinned to the surface as the tilt angle increased. The PDMS and SNAP alone did not reduce the substrate's surface tension enough to allow sliding. On the other hand, both the NE-swelled samples and the 2%EG-swelled samples demonstrated low sliding angles between 11.00° ± 1.12° and 12.78° ± 0.97°, suggesting that the eugenol incorporation does not affect the initial slippery behavior of the NE (Fig. 4A). These sliding angles are comparable to previously reported liquid-infused NO-releasing silicone rubber systems,^16,33,35^ which achieve anti-fouling behavior using either inert silicone oil lubricants or unloaded aliphatic-based nanoemulsions. As the majority of the NE is composed of *n*-hexadecane, the overall surface tension of the lubricant layer is low (27.47 mN m^−1^),^44^ especially compared to that of water (72.80 mN m^−1^).^45^ This difference in surface tension allows for a water droplet to essentially sit on top of the lubricant layer and simply slide off the surface.

Many medical devices are exposed to biological environments for longer than the first few critical hours after insertion or implantation. Therefore, maintaining device functionality and integrity throughout the insertion is vital. The slippery behavior of the SNAP-2%EG and the controls was thus evaluated over 24 h. After the initial sliding angle of the samples was measured, the samples were incubated in a dynamic physiological environment (10 mM PBS with 100 μM EDTA, 37 °C, 150 rpm) for 24 h. After removing the samples from the physiological environment and gently removing the excess PBS, the samples were allowed to replenish the surface lubricant for 1 min before measuring the sliding angle. Although the initial sliding angle measurements indicate initial slippery behavior, incubation in a dynamic physiological environment depleted some of the surface lubricant (Fig. 4B). Even though the depletion resulted in a significant increase in sliding angles across the NE-swelled and 2%EG-swelled samples, they still remained below 20° and were considered slippery. Similar trends in lubricant depletion have been reported for liquid-infused surfaces exposed to dynamic physiological conditions.^35^ However, the NE-infused matrix's ability to maintain slippery behavior during this critical early exposure window is promising because the first 24 h after device insertion or implantation are the most critical for preventing infection and thrombosis. For short-term applications, such as short-term catheterization, the sliding angle remains below 20° for 7 d (Fig. S5).

It is important to note that these tests were conducted under mild dynamic conditions that do not fully represent shear forces in blood flow. The mild conditions were an initial assessment of surface stability for this proof-of-concept material and were used to mimic experimental conditions used in other biological studies, such as the antibacterial assessment. While not a direct mimic of shear conditions, such shaking conditions enabled agitation on the surface to deplete some of the lubricant. For more controlled shear-flow conditions, using flow chambers to analyze surface stability would be advantageous.

### 3.4. Eugenol loading

As the major compound in clove oil, eugenol is highly soluble in organic solvents, such as *n*-hexadecane, compared to aqueous solvents. Eugenol has a very low water solubility,^25,27^ lending itself to near-immediate phase separation at higher concentrations. However, indwelling medical devices are typically subjected to physiological environments that more closely resemble aqueous conditions rather than organic solvents. Therefore, eugenol initial loading and diffusion from the NE were evaluated over 24 h.

**Fig. 5:**
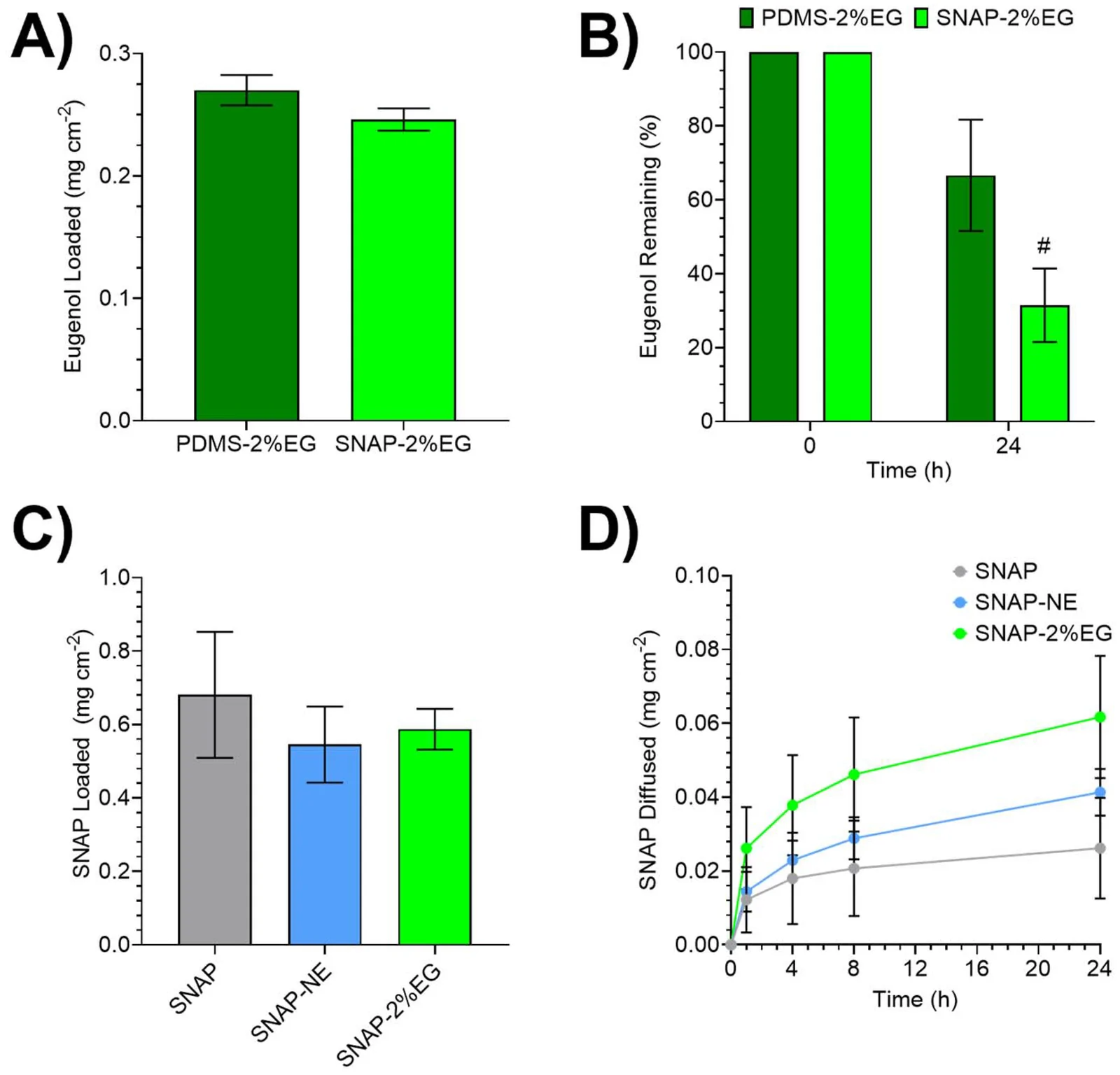
(A) Eugenol loaded into PDMS-2%EG and SNAP-2%EG. Eugenol only made up 2% v/v of the total NE oil phase. (B) Eugenol remaining within PDMS-2%EG and SNAP-2%EG after incubation in physiological conditions for 24 h (# *p* < 0.05 *vs*. PDMS-2%EG at hour 24). (C) SNAP loading and (D) diffusion from SNAP-impregnated materials. The amount of SNAP loaded into each sample were not significantly different, indicating that NE-infusion does not affect the SNAP reservoir loaded into the polymer matrix. In addition, the amount of SNAP diffused from the surfaces over 24 h was less than 10% of the total amount loaded (*n* = 4).

The amount of eugenol loaded into the 2%EG-containing samples was around 0.27 ± 0.01 mg cm^−2^ and 0.25 ± 0.01 mg cm^−2^ for PDMS-2%EG and SNAP-2%EG, respectively (Fig. 5A), indicating that the presence of SNAP does not affect eugenol swelling (Fig. S6 and S7). Incorporation of eugenol within the nanoemulsion lubricant phase provides a diffusion-limited reservoir that supports sustained antimicrobial activity while preserving cytocompatibility. Notably, eugenol in this system functions as a complementary bioactive agent, augmenting NO-mediated antibacterial and platelet-modulating effects rather than serving as the sole therapeutic component.

After incubation in physiological conditions (PBS w/EDTA @ 37 °C) for 24 h, the eugenol remaining within the samples was also quantified. It was found that the percent of eugenol remaining within the substrate after incubation in physiological conditions was 66.62% and 31.47% for PDMS-2%EG and SNAP-2%EG, respectively (Fig. 5B). The significant difference in the amount of eugenol remaining in the PDMS-2%EG and SNAP-2%EG samples can be attributed to SNAP diffusing from the matrix, thereby facilitating some eugenol release from the lubricant as well. Although much of the eugenol was released in the first 24 h, which aids in combating initial infection, the remaining amount of eugenol is expected to slowly deplete over the following days to maintain the antibacterial efficacy for short-term devices. As the starting concentration of eugenol loaded into the substrates was relatively low, the samples are not expected to elicit cytotoxic effects on mammalian cells while being able to maintain their antibacterial and anti-platelet potential.

### 3.5. SNAP loading and leaching

The NO donor SNAP can be incorporated into polymers by facile methods, such as solvent impregnation or polymer blending.^19,33,46^ With the polymer blending technique, there is an exact amount of SNAP that is incorporated into the polymer solution; however, with the impregnation technique, the final amount of SNAP must be determined through a SNAP loading experiment. The samples containing SNAP were incubated in THF to promote the removal of SNAP from the polymer matrix, and the absorbance of the leachate was measured. The amounts of SNAP loaded in SNAP, SNAP-NE, and SNAP-2%EG were not significantly different, with averages ranging from 0.55 ± 0.10 mg cm^−2^ to 0.68 ± 0.17 mg cm^−2^ (Fig. 5C). This demonstrates that incorporating NE or 2%EG does not significantly affect the SNAP reservoir within the polymeric matrix.

Because the SNAP was incorporated into the PDMS *via* solvent impregnation, the SNAP within the PDMS is present only in a predominantly crystalline state; it is not covalently bound to the polymeric backbone.^18^ Therefore, the SNAP molecule can also diffuse from the matrix. At high concentrations, the level of SNAP diffusion from the polymer device could be detrimental or toxic to the surrounding tissue. However, with samples incubated under physiological conditions (PBS w/EDTA @ 37 °C), the highest diffusion rate occurred in the first hour and then slowly stabilized over 24 h. By 24 h, SNAP, SNAP-NE, and SNAP-2%EG all exhibited low levels of SNAP leaching (0.03 ± 0.01, 0.04 ± 0.01, and 0.06 ± 0.02 mg cm^−2^, respectively) and were not significantly different from each other (Fig. 5D). Although the average SNAP diffusion from SNAP-NE and SNAP-2%EG was higher than that of SNAP, most likely due to NE swelling allowing a less condensed polymer network, thereby increasing SNAP diffusivity through the matrix, the amount leached was less than 10% of the total SNAP loaded into each sample type.

### 3.6. NO release profile

As an endogenous free radical gasotransmitter molecule, NO can freely and easily diffuse through polymer matrices and lubricant layers.^16,21,47^ As an RSNO, the SNAP impregnated into the PDMS will readily sever its S–NO bond through hydrolysis, heat, light, or metal ion catalysis.^48,49^ However, with the addition of a hydrophobic lubricant barrier layer, rapid release of NO is prevented due to the protection of the lubricant. The rate at which NO was released from the SNAP-incorporated samples was measured using gold-standard chemiluminescence detection with a Nitric Oxide Analyzer (NOA) over 24 h.

**Fig. 6:**
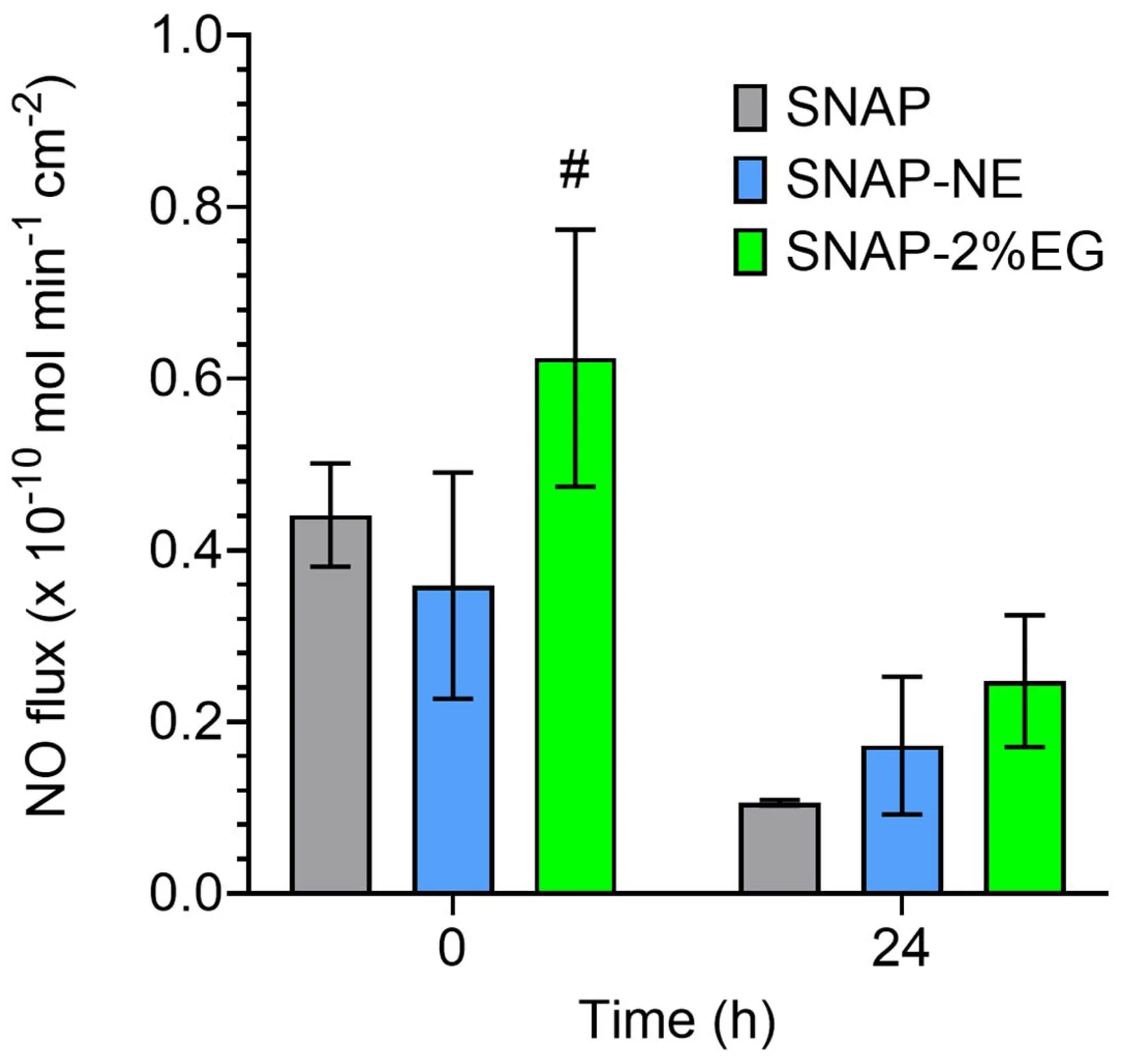
NO release from the SNAP-impregnated samples. All samples released near physiological levels of NO in the first 24 h of measurement (*n* ≥ 3, # *p* < 0.05 *vs*. SNAP-NE).

On day 0, the samples released NO between 0.36 × 10^−10^ mol min^−1^ cm^−2^ and 0.62 × 10^−10^ mol min^−1^ cm^−2^ (Fig. 6). While these values are on the cusp of the physiological range (0.5–4 × 10^−10^ mol min^−1^ cm^−2^),^24^ all sample groups continued to release NO for 24 h, at levels between 0.11 × 10^−10^ mol min^−1^ cm^−2^ and 0.25 × 10^−10^ mol min^−1^ cm^−2^. This is comparable to the NO release reported in the literature for liquid-infused surfaces.^16^ The NO fluxes observed here fall near physiologically relevant ranges while avoiding excess donor leaching. The maintenance of sustained NO in the presence of a hydrophobic lubricant layer highlights the compatibility of nanoemulsion infusion with established NO-delivery strategies. The average NO flux of SNAP-2%EG is higher than that of SNAP, most likely due to the nanoemulsion swelling. During the 4 h NE infusion, the swollen samples showed swelling of the polymer network, allowing NE to penetrate and establish a reservoir within the matrix. This temporary expansion of the crosslinked PDMS chains potentially allows for increased SNAP diffusion, which is corroborated by the amount of SNAP leached from the substrate into the physiological media (Fig. 5C), thereby increasing the overall NO release. Despite the lower concentrations of NO release in the 24 h, these levels (ranging from picomolar levels^50^ to 500 nM (ref. 51)) have previously been reported to demonstrate antibacterial efficacy despite sub-physiological release. Therefore, these materials would still be suitable for antibacterial substrates.

### 3.7. *In vitro* antibacterial efficacy evaluation

Bacterial infection on the surface of an indwelling device is one of the prevalent causes of clinical concern because of the potential for morbidity and mortality if left untreated.^2^ Antibiotic administration is currently the gold standard among healthcare professionals for prophylaxis and post-emergence treatment; however, bacteria become less susceptible to antibiotics as they mature and develop extracellular polymeric substances (EPS).^3,4^ The maturation and protection that the EPS provides make attacking the infection and possible biofilm that much more difficult. Therefore, the need for alternative strategies to combat bacterial infection is essential to keep pace with bacterial evolution. Bioactive and slippery surfaces, like SNAP-2%EG, offer multiple mechanisms to prevent bacterial infection, ranging from actively killing bacteria through NO release and/or eugenol diffusion to passively preventing bacteria from adhering at all.

**Fig. 7:**
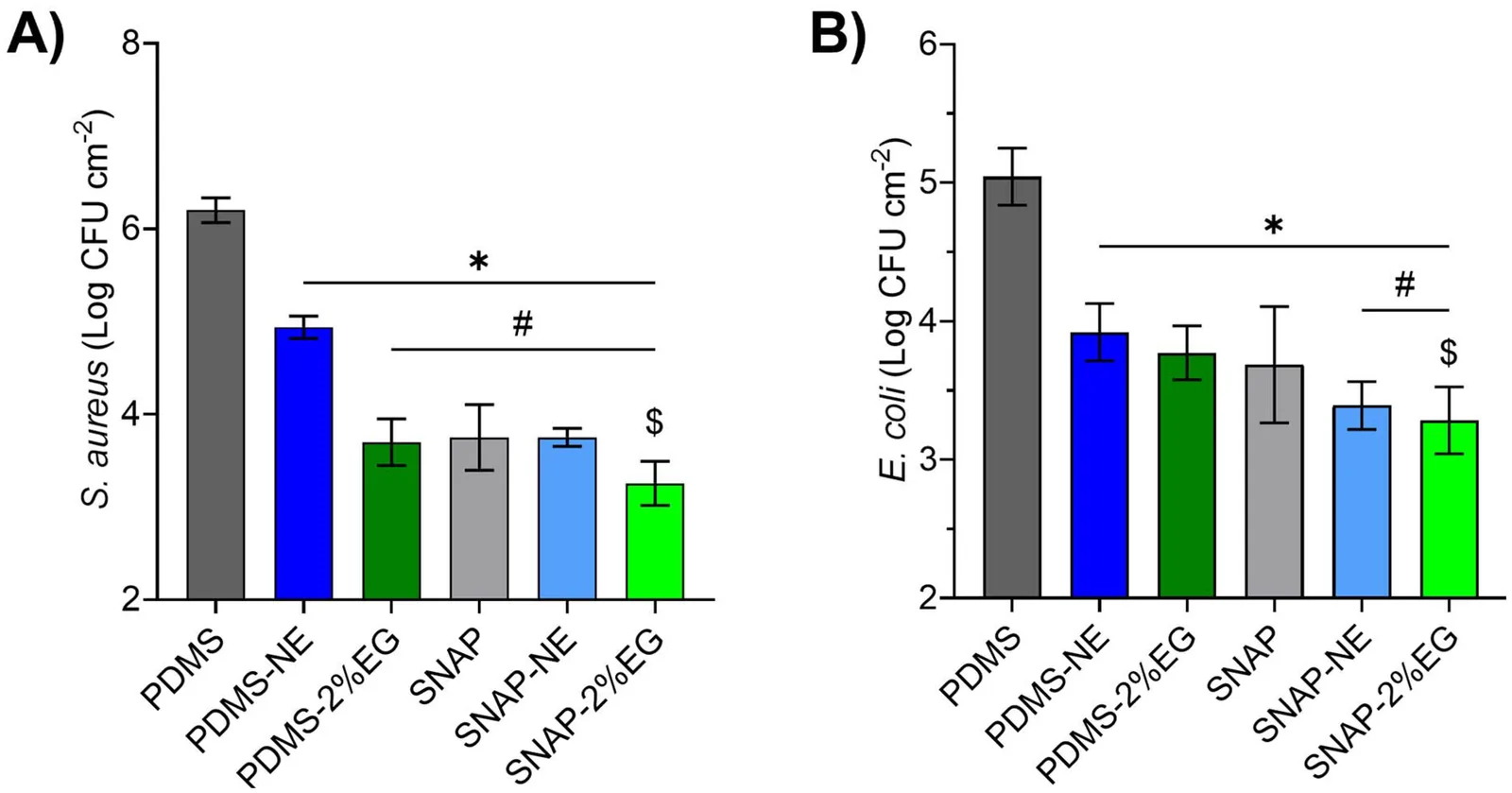
Reduction of bacterial adhesion against (A) *S. aureus* (*n* = 4, * *p* < 0.05 *vs*. PDMS, # *p* < 0.05 *vs*. PDMS-NE, $ *p* < 0.05 *vs*. SNAP and SNAP-NE) and (B) *E. coli* (*n* = 4, * *p* < 0.05 *vs*. PDMS, # *p* < 0.05 *vs*. PDMS-NE, $ *p* < 0.05 *vs*. PDMS-2%EG). The SNAP-2%EG was able to reduce viable *S. aureus* by 2.95 log and *E. coli* by 1.76 log.

The antibacterial efficacy of SNAP-2%EG and its controls was determined against the Gram-positive *S. aureus* and the Gram-negative *E. coli* in a 24 h adhesion assay, given their relevance to clinical infections.^52–54^ The 24 h assay was chosen to evaluate the SNAP-2%EG material during the critical infection window after device implantation or insertion, when preventing bacterial adhesion is especially crucial. Prior to bacterial solution exposure, the samples were UV sterilized, which did not affect the NO release capability nor the amount of eugenol swelled into the experimental samples (Fig. S8). While the control PDMS did not exhibit any antibacterial activity, the SNAP-2%EG material reduced *S. aureus* and *E. coli* viability by 2.95 log and 1.76 log, respectively, compared to PDMS, demonstrating broad-spectrum efficacy against both Gram-positive and Gram-negative bacteria (Fig. 7A and B). The production of nitrosative and oxidative species *via* NO led to bacterial membrane disruption, DNA alteration, and lipid peroxidation.^55^ Eugenol causes similar bacterial membrane disruption, in which the compound's hydroxyl group is suggested to bind to membrane proteins, altering membrane permeability and resulting in cellular leakage and lysis.^29^ The nearly 3-log reduction against *S. aureus* is comparable to or exceeds reductions reported for NO-releasing slippery surfaces and NE-infused platforms lacking hydrophobic bioactive agents,^34,35^ highlighting the advantage of combining NO-releasing with eugenol-loaded lubricant reservoirs. It is important to note that the SNAP-2%EG performed more effectively against the Gram-positive *S. aureus* compared to the Gram-negative *E. coli*. Previous reports have shown that NO's efficacy is slightly reduced against Gram-negative strains due to the lipopolysaccharide outer membrane.^56^ Because *S. aureus* lacks the additional protective layer, it is more susceptible to NO's antibacterial mechanisms.

Despite differences in efficacy, the combination group SNAP-2%EG was especially effective compared with its controls. These nearly 3-log and 2-log reductions in *S. aureus* and *E. coli* are attributed to the active components NO and eugenol, as well as to the passive slippery lubricant layer, which prevents bacteria from adhering. Compared to previous NO-releasing, nanoemulsion-infused platforms,^35^ the SNAP-2%EG has a different composition and an overall lower NO release. However, these active mechanisms, in combination with the low surface tension of the slippery lubricant layer, performed comparably to previous NO-releasing and slippery surfaces,^16,33,35^ specifically with nearly 3 log reduction of *S. aureus* and over 1.5 log reduction of *E. coli*.

It is important to note that the samples tested here do not fully represent the curvature of an implantable medical device, such as an intravascular catheter, which can add complexity under dynamic conditions. As such, silicone rubber catheter samples were prepared similarly to the Sylgard 184 PDMS samples. The anti-fouling potential and antibacterial efficacy were assessed in a similar fashion. The catheter samples exhibited comparable sliding angles below 20°, suggesting that the slippery lubricant was evenly distributed on the surface. The PDMS-2%EG and SNAP-2%EG samples also exhibited over 2 log reduction of *S. aureus* adhesion (Fig. S9). These results suggest that the material design can be translated to different polymer architectures without compromising the efficacy.

### 3.8. *In vitro* biocompatibility assessment

#### 3.8.1. Cell cytocompatibility

Commercial medical devices are rigorously tested to assess their biocompatibility with mammalian cells, ensuring no adverse effects or cell death in the surrounding environment. According to ISO 10993-5, the cytocompatibility threshold is >70% relative cell viability after exposure to the test material.^40^ High concentrations of eugenol or high amounts of NO release have previously been reported to be cytotoxic to mammalian cells,^57–59^ and could be a cause for discontinuation of biomedical use. Therefore, L-929 mouse fibroblast cells were cultured and indirectly exposed to SNAP-2%EG and its controls *via* leachate exposure.

**Fig. 8:**
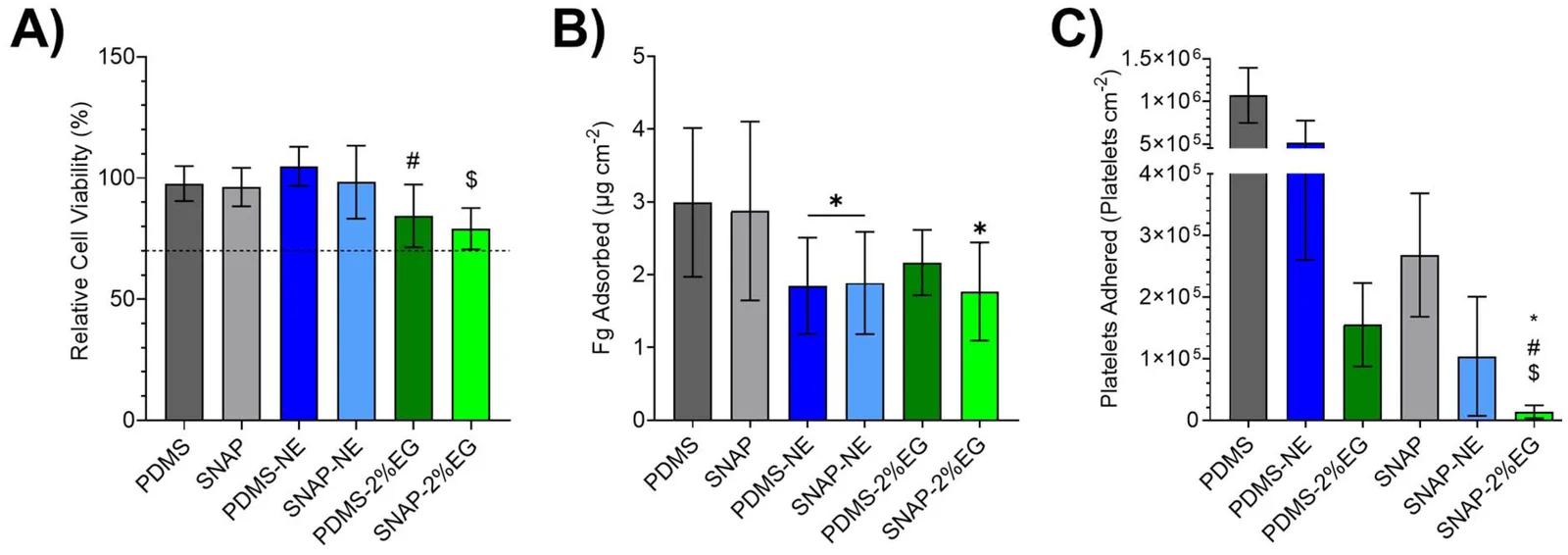
(A) Cytocompatibility of SNAP-2%EG and its controls (*n* = 4, # *p* < 0.05 *vs*. PDMS-NE, $ *p* < 0.05 *vs*. all other sample types except PDMS-2%EG). While the relative cell viability of the cells exposed to SNAP-2%EG was slightly reduced compared to the other sample types, it remained above 70% and was suitable for use according to ISO standards. Reduction of (B) fibrinogen adsorbed and (C) platelets adhered to the sample surfaces (*n* = 8, * *p* < 0.05 *vs*. PDMS, # *p* < 0.05 *vs*. PDMS-NE, $ *p* < 0.05 *vs*. SNAP). The NO release from the SNAP-2%EG did not significantly increase the amount of Fg adsorbed onto the surface compared to the non-NO-releasing sample, indicating that the slippery lubricant layer mitigated the effect from NO. In addition to the reduction of adsorbed Fg, SNAP-2%EG demonstrated over a 1 log reduction of adhered platelets, showing that the combination of NO release, eugenol diffusion, and slippery behavior was more effective than any one single strategy.

After 24 h of exposure to the leachates, the cells remained viable. Incubation with the MTT working solution produced abundant formazan crystals, indicative of cellular metabolic activity. All sample groups exhibited >70% relative cell viability, which exceeds the ISO threshold for cytocompatibility of medical devices (Fig. 8A). It is important to note that although eugenol from PDMS-2%EG and SNAP-2%EG diffused out of the sample, the resulting concentration was insufficient to induce cell death at cell viabilities below 70%. Therefore, even at low eugenol incorporation levels, these materials remain suitable for biomedical applications.

#### 3.8.2. Prevention of fibrinogen adsorption

One of the first interactions that blood-contacting devices have when indwelling is protein surface interactions. When the foreign material contacts the blood, the almost immediate and irreversible adsorption of fibrinogen (Fg) on the surface and the subsequent conversion of fibrinogen to fibrin aids thrombus formation.^60^ The bound protein allows for passing platelets and red blood cells to activate, aggregate, and form a clot.^60^ This protein accumulation not only attracts blood components to aggregate but also promotes bacterial adhesion, leading to infection developing on the surface of the device.^61^ Although NO-releasing materials have excellent antibacterial and platelet-modulating properties, proteins tend to adsorb more to these types of surfaces.^62^ Therefore, a combination of anti-fouling strategies must be used with NO-releasing surfaces to comprehensively address the biofouling problem.

The prevalent blood plasma protein fibrinogen, a vital component of the coagulation cascade, was used as the model protein in this study. While the control PDMS and SNAP exhibited higher levels of adsorbed Fg (2.99 ± 1.02 μg cm^−2^ and 2.87 ± 1.23 μg cm^−2^, respectively), all of the NE-swelled samples demonstrated a reduction in Fg between 27.63% and 40.93% (Fig. 8B). The combination of NO release and slippery surfaces, SNAP-NE and SNAP-2%EG, did not have a significant increase in Fg adsorbed compared to their controls, indicating that the slippery surface was effective at mitigating the NO release's nature to enhance Fg adsorption.

#### 3.8.3. Prevention of platelet adhesion

Blood-contacting devices, such as short-term catheters or extracorporeal life support systems, are exposed to the complex environment of blood: blood cells, plasma proteins, and platelets. The complexity of this environment poses a major challenge for the onset of thrombogenic events by foreign materials, which can trigger the signaling cascade of clotting factors and induce platelet plug formation.^60^ The irreversible adsorption of plasma proteins (*e.g.*, fibrinogen) to the platelet plug encapsulates other blood components like red blood cells and exacerbates the thrombus, leading to occlusion and failure of the device. The current method of preventing or treating thrombosis is the systemic administration of anticoagulants; however, adverse side effects like excessive bleeding or platelet consumption remain an issue.^11–13^

Nitric oxide has been previously studied for its platelet-modulating effects. Specifically, NO alone suppresses platelet activation, thereby significantly reducing platelet adhesion to substrates.^63,64^ However, the inclusion of an anti-fouling strategy can further reduce platelet adhesion by creating a physical barrier between the platelets and the material surface. In this case, the slippery behavior of the NE-swelled samples is attributed to their low surface tension, which prevents platelets from adhering. In addition to the anti-fouling capability of the NE, the eugenol-incorporated samples have an additional advantage: eugenol itself has platelet-modulating effects. Eugenol has been reported to prevent platelet activation by suppressing key signaling pathways, resulting in decreased intracellular calcium levels and reduced platelet aggregation.^30,31^ This, in combination with NO and anti-fouling behavior, was expected to reduce platelet counts more than its controls.

While the control PDMS had 1.07 × 10^6^ platelets cm^−2^ adhered to the surface, SNAP-2%EG significantly reduced the number to 1.38 × 10^4^ platelets cm^−2^, corresponding to a 98.71% reduction (Fig. 8C). Compared to the previous literature with NO-releasing and NE-infused substrates, which demonstrated a 89.92% reduction of adhered platelets,^35^ the SNAP-2%EG material was able to reduce platelet adhesion by nearly 2 log. The combination of the two bioactive components with the passive slippery surface enhanced the reduction of thrombogenic events compared to materials with just one bioactive component. All control groups reduced platelet adhesion with reductions between 51.62% and 90.30%, demonstrating that the individual strategies were effective to some extent (Fig. 8C). While previous NO-releasing materials have demonstrated effective platelet suppression but increased Fg adsorption, and slippery surfaces have reduced adhesion without active modulation, the combination strategy employed here overcomes these individual limitations. The multifunctional surface, which incorporates NO release, eugenol diffusion, and slippery characteristics, was significantly more effective at deterring platelet adhesion than any single strategy alone. This enhanced performance aligns with previous reports indicating that NO-based substrates, when paired with additional anti-fouling agents, can achieve greater reductions in biofouling.^16,35^ Although these assays provide early indicators for thrombus reduction, they do not fully represent complex thrombotic processes. Further investigation is necessary to fully understand the material's antithrombotic potential.

## 4. Conclusions

Clinical challenges such as infection and thrombosis remain critical issues for healthcare professionals, especially amid the rise of antibiotic resistance and the adverse effects of anticoagulants. Without alternative treatments or strategies to address these challenges, infection, platelet aggregation, antibiotic resistance, and adverse anticoagulant effects will continue to plague healthcare settings.

It was hypothesized that integrating a hydrophobic antimicrobial within a nanoemulsion lubricant reservoir, combined with controlled NO release, would enable enhanced passive and active mitigation of infection and platelet aggregation. This study demonstrates a multifunctional surface modification strategy that combines slippery NE infusion with controlled NO release and eugenol delivery to mitigate the onset of platelet adhesion and infection on PDMS-based medical device materials. The *n*-hexadecane NEs successfully swelled into the SNAP-impregnated polymer network within 4 h, forming a stable lubricant reservoir without diminishing SNAP loading. These NE-infused surfaces retained low sliding angles below 20° even after 24 h in dynamic physiological conditions, confirming durable slippery behavior. Both eugenol and SNAP exhibited controlled, low-level diffusion, while NO fluxes remained within ranges known to induce antibacterial activity. Together, these characteristics yielded a material platform that maintained cytocompatibility with relative cell viability >70% and reduced Fg adsorption and platelet adhesion by 39% and 98%, respectively. In addition, the combined active and passive antibacterial and anti-fouling mechanisms of the SNAP-2%EG surface achieved 2.95 log reductions in *S. aureus* and 1.76 log reductions in *E. coli*. Relative to previously reported NO-releasing polymers or slippery liquid-infused surfaces, this multifunctional platform uniquely integrates dual bioactive delivery with sustained anti-fouling behavior, achieving comparable or improved antibacterial and anti-platelet performance without additional surface chemistries, multilayer fabrication, or loss of cytocompatibility.

This work highlights a unique combination of a second bioactive agent, eugenol, in the lubricant phase of a slippery nanoemulsion infusion into a NO-releasing silicone rubber matrix. It provides a surface-mediated controlled release of one bioactive while also introducing direct bioactivity to the slippery lubricant medium. Unlike previously reported lubricant-infused slippery surfaces, this work offers the possibility of contact-antibacterial effects and/or platelet-modulating properties, rather than just passive prevention of adhesion. This work introduces a more potent shift from a surface-confined bioactivity (*i.e.*, the NO-releasing substrate) to a bioactive liquid interface (*i.e.*, eugenol-loaded NE). This material would be effective in reducing financial burden and material use because it would reduce the number of medical devices that would need to be replaced due to failure. It is important to note that the primary objective of this study was to establish the multifunctional surface design and systematically evaluate its performance under controlled conditions. The exclusion of *in vivo* validation in this work is certainly a limitation, but one intended to highlight the material characterization itself. However, looking ahead, evaluating the SNAP-2%EG material's ability to address biofilm accumulation and thrombosis in dynamic models, such as a 3 d dripflow bioreactor or a 4 h extracorporeal circuit (ECC) rabbit model, is paramount to fully investigate the material's antibacterial performance and thromboresistance *in vivo*. Altogether, these findings highlight the potential of bioactive, slippery NE-infused NO-releasing surfaces as a promising approach for preventing early-stage infection and thrombosis on indwelling medical devices.

## Author contributions

Grace H. Nguyen: conceptualization, data curation, formal analysis, investigation, methodology, validation, visualization, writing – original draft, writing – review & editing. Everett Grizzle: data curation, formal analysis, investigation, methodology, validation, visualization, writing – review & editing. Elizabeth J. Brisbois: project administration, data curation, funding acquisition, resources, supervision, writing – review & editing.

## Conflicts of interest

The authors declare the following competing financial interest(s): Elizabeth J. Brisbois is a cofounder and maintains a financial interest in a startup company investigating nitric oxide as a biomedical therapeutic for medical devices.

## Supplementary Material

BM-014-D6BM00767H-s001

## Data Availability

Data will be made available upon request. Supplementary information (SI) is available. See DOI: https://doi.org/10.1039/d6bm00767h.

## References

[cit1] Vanepps J. S., Younger J. G. (2016). Implantable Device-Related Infection. Shock.

[cit2] Bryers J. D. (2008). Medical biofilms. Biotechnol. Bioeng..

[cit3] Stewart P. S., William Costerton J. (2001). Antibiotic, resistance of bacteria in biofilms. Lancet.

[cit4] Mancuso G., Midiri A., Gerace E., Biondo C. (2021). Bacterial Antibiotic Resistance: The Most Critical Pathogens. Pathogens.

[cit5] Ceri H., Olson M. E., Stremick C., Read R. R., Morck D., Buret A. (1999). The Calgary Biofilm Device: New Technology for Rapid Determination of Antibiotic Susceptibilities of Bacterial Biofilms. J. Clin. Microbiol..

[cit6] Jaffer I. H., Fredenburgh J. C., Hirsh J., Weitz J. I. (2015). Medical device-induced thrombosis: what causes it and how can we prevent it?. J. Thromb. Haemostasis.

[cit7] Jaffer I. H., Weitz J. I. (2019). The blood compatibility challenge. Part 1: Blood-contacting medical devices: The scope of the problem. Acta Biomater..

[cit8] Holdsworth M., Welch S., Borrego M., Spyropoulos A., Mahan C. (2011). Deep-vein thrombosis: A United States cost model for a preventable and costly adverse event. Thromb. Haemostasis.

[cit9] Smith D., Murauski J. (2017). Pulmonary Embolism in the Postanesthesia Care Unit: A Case Study. J. PeriAnesth. Nurs..

[cit10] Salzman E. W., Rosenberg R. D., Smith M. H., Lindon J. N., Favreau L. (1980). Effect of Heparin and Heparin Fractions on Platelet Aggregation. J. Clin. Invest..

[cit11] Rettie A. E. (2006). The Pharmocogenomics of Warfarin: Closing in on Personalized Medicine. Mol. Interv..

[cit12] Yeh R. W., Jang I.-K. (2006). Argatroban: Update. Am. Heart J..

[cit13] Freedman M. D. (1992). Oral Anticoagulants: Pharmacodynamics, Clinical Indications and Adverse Effects. J. Clin. Pharmacol..

[cit14] Wysowski D. K., Nourjah P., Swartz L. (2007). Bleeding Complications With Warfarin Use. Arch. Intern. Med..

[cit15] Brisbois E. J., Kim M., Wang X., Mohammed A., Major T. C., Wu J., Brownstein J., Xi C., Handa H., Bartlett R. H. (2016). *et al.*, Improved Hemocompatibility of Multilumen Catheters via Nitric Oxide (NO) Release fromS-Nitroso-N-acetylpenicillamine (SNAP) Composite Filled Lumen. ACS Appl. Mater. Interfaces.

[cit16] Goudie M. J., Pant J., Handa H. (2017). Liquid-infused nitric oxide-releasing (LINORel) silicone for decreased fouling, thrombosis, and infection of medical devices. Sci. Rep..

[cit17] Griffin L., Douglass M., Goudie M., Hopkins S. P., Schmiedt C., Handa H. (2022). Improved Polymer Hemocompatibility for Blood-Contacting Applications via S-Nitrosoglutathione Impregnation. ACS Appl. Mater. Interfaces.

[cit18] Hopkins S. P., Pant J., Goudie M. J., Schmiedt C., Handa H. (2018). Achieving Long-Term Biocompatible Silicone via Covalently ImmobilizedS-Nitroso-N-acetylpenicillamine (SNAP) That Exhibits 4 Months of Sustained Nitric Oxide Release. ACS Appl.
Mater. Interfaces.

[cit19] Mondal A., Douglass M., Hopkins S. P., Singha P., Tran M., Handa H., Brisbois E. J. (2019). Multifunctional S-Nitroso-N-acetylpenicillamine-Incorporated Medical-Grade Polymer with Selenium Interface for Biomedical Applications. ACS Appl. Mater. Interfaces.

[cit20] Sapkota A., Mondal A., Chug M. K., Brisbois E. J. (2023). Biomimetic catheter surface with dual action NO-releasing and generating properties for enhanced antimicrobial efficacy. J. Biomed. Mater. Res., Part A.

[cit21] Zhang H., Annich G. M., Miskulin J., Osterholzer K., Merz S. I., Bartlett R. H., Meyerhoff M. E. (2002). Nitric oxide releasing silicone rubbers with improved blood compatibility: preparation, characterization, and in vivo evaluation. Biomaterials.

[cit22] Feit C. G., Chug M. K., Brisbois E. J. (2019). Development of S-Nitroso-N-Acetylpenicillamine Impregnated Medical Grade Polyvinyl Chloride for Antimicrobial Medical Device Interfaces. ACS Appl. Bio Mater..

[cit23] Devine R., Goudie M. J., Singha P., Schmiedt C., Douglass M., Brisbois E. J., Handa H. (2020). Mimicking the Endothelium: Dual Action Heparinized Nitric Oxide Releasing Surface. ACS Appl. Mater. Interfaces.

[cit24] Vaughn M. W., Kuo L., Liao J. C. (1998). Estimation of nitric oxide production and reactionrates in tissue by use of a mathematical model. Am. J. Physiol.: Heart Circ. Physiol..

[cit25] Kowalewska A., Majewska-Smolarek K. (2023). Eugenol-Based Polymeric Materials—Antibacterial Activity and Applications. Antibiotics.

[cit26] Ulanowska M., Olas B. (2021). Biological Properties and Prospects for the Application of Eugenol-A Review. Int. J. Mol. Sci..

[cit27] Khalil A. A., Rahman U. u., Khan M. R., Sahar A., Mehmood T., Khan M. (2017). Essential oil eugenol: sources, extraction techniques and nutraceutical perspectives. RSC Adv..

[cit28] Devi K. P., Nisha S. A., Sakthivel R., Pandian S. K. (2010). Eugenol (an essential oil of clove) acts as an antibacterial agent against Salmonella typhi by disrupting the cellular membrane. J. Ethnopharmacol..

[cit29] Jeyakumar G. E., Lawrence R. (2021). Mechanisms of bactericidal action of Eugenol against Escherichia coli. J. Herb. Med..

[cit30] Huang W.-C., Shu L.-H., Kuo Y.-J., Lai K. S.-L., Hsia C.-W., Yen T.-L., Hsia C.-H., Jayakumar T., Yang C.-H., Sheu J.-R. (2024). Eugenol Suppresses Platelet Activation and Mitigates Pulmonary Thromboembolism in Humans and Murine Models. Int. J. Mol. Sci..

[cit31] Saeed S. A., Simjee R. U., Shamim G., Gilani A. H. (1995). Eugenol: a dual inhibitor of platelet-activating factor and arachidonic acid metabolism. Phytomedicine.

[cit32] Wong T.-S., Kang S. H., Tang S. K. Y., Smythe E. J., Hatton B. D., Grinthal A., Aizenberg J. (2011). Bioinspired self-repairing slippery surfaces with pressure-stable omniphobicity. Nature.

[cit33] Chug M. K., Feit C., Brisbois E. J. (2019). Increasing the Lifetime of Insulin Cannula with Antifouling and Nitric Oxide Releasing Properties. ACS Appl. Bio Mater..

[cit34] Agarwal H., Polaske T. J., Sánchez-Velázquez G., Blackwell H. E., Lynn D. M. (2021). Slippery nanoemulsion-infused porous surfaces (SNIPS): anti-fouling coatings that can host and sustain the release of water-soluble agents. Chem. Commun..

[cit35] Nguyen G. H., Garren M., Wu Y., Mondal A., Handa H., Brisbois E. J. (2025). Multifunctional slippery nanoemulsion-infused porous nitric oxide-releasing surfaces. J. Colloid Interface Sci..

[cit36] Nguyen G. H., Sapkota A., Brisbois E. J. (2025). Dual-action prevention of adherent and non-adherent biofouling via slippery, nitric oxide-releasing nanoemulsion-infused porous surfaces. Biomater. Sci..

[cit37] Chipinda I., Simoyi R. H. (2006). Formation and Stability of a Nitric Oxide Donor: S-Nitroso-N-acetylpenicillamine. J. Phys. Chem. B.

[cit38] Brisbois E. J., Major T. C., Goudie M. J., Bartlett R. H., Meyerhoff M. E., Handa H. (2016). Improved hemocompatibility of silicone rubber extracorporeal tubing via solvent swelling-impregnation of S-nitroso-N-acetylpenicillamine (SNAP) and evaluation in rabbit thrombogenicity model. Acta Biomater..

[cit39] Hassan P. A., Rana S., Verma G. (2015). Making Sense of Brownian Motion: Colloid Characterization by Dynamic Light Scattering. Langmuir.

[cit40] International Organization for Standardization , ISO 10993-5: 2009 Biological evaluation of medical devices - Part 5: Test for in vitro cytotoxicity, 2009

[cit41] International Organization for Standardization , ISO 10993-4: 2017 Biological evaluation of medical devices—Part 4: Selection of tests for interactions with blood, International Organizational for Standardization Geneve, 2017

[cit42] Jaiswal M., Dudhe R., Sharma P. K. (2015). Nanoemulsion: an advanced mode of drug delivery system. 3 Biotech.

[cit43] Wu H., Ramachandran C., Weiner N. D., Roessler B. J. (2001). Topical transport of hydrophilic compounds using water-in-oil nanoemulsions. Int. J. Pharm..

[cit44] Klein T., Yan S., Cui J., Magee J. W., Kroenlein K., Rausch M. H., Koller T. M., Fröba A. P. (2019). Liquid Viscosity and Surface Tension of n-Hexane, n-Octane, n-Decane, and n-Hexadecane up to 573 K by Surface Light Scattering. J. Chem. Eng. Data.

[cit45] Hauner I. M., Deblais A., Beattie J. K., Kellay H., Bonn D. (2017). The Dynamic Surface Tension of Water. J. Phys. Chem. Lett..

[cit46] Brisbois E. J., Davis R. P., Jones A. M., Major T. C., Bartlett R. H., Meyerhoff M. E., Handa H. (2015). Reduction in thrombosis and bacterial adhesion with 7 day implantation of S-nitroso-N-acetylpenicillamine (SNAP)-doped Elast-eon E2As catheters in sheep. J. Mater. Chem. B.

[cit47] Ren H., Bull J. L., Meyerhoff M. E. (2016). Transport of Nitric Oxide (NO) in Various Biomedical grade Polyurethanes: Measurements and Modeling Impact on NO Release Properties of Medical Devices. ACS Biomater. Sci. Eng..

[cit48] Douglass M. E., Goudie M. J., Pant J., Singha P., Hopkins S., Devine R., Schmiedt C. W., Handa H. (2019). Catalyzed Nitric Oxide Release via Cu Nanoparticles Leads to an Increase in Antimicrobial Effects and Hemocompatibility for Short-Term Extracorporeal Circulation. ACS Appl. Bio Mater..

[cit49] Chug M. K., Brisbois E. J. (2022). Smartphone compatible nitric oxide releasing insert to prevent catheter-associated infections. J. Controlled Release.

[cit50] Estes Bright L. M., Griffin L., Mondal A., Hopkins S., Ozkan E., Handa H. (2022). Biomimetic gasotransmitter-releasing alginate beads for biocompatible antimicrobial therapy. J. Colloid Interface Sci..

[cit51] Barraud N., Hassett D. J., Hwang S.-H., Rice S. A., Kjelleberg S., Webb J. S. (2006). Involvement of Nitric Oxide in Biofilm Dispersal of *Pseudomonas aeruginosa*. J. Bacteriol..

[cit52] Sharma G., Sharma S., Sharma P., Chandola D., Dang S., Gupta S., Gabrani R. (2016). Escherichia coli biofilm: development and therapeutic strategies. J. Appl. Microbiol..

[cit53] Weinstein R. A., Darouiche R. O. (2001). Device-Associated Infections: A Macroproblem that Starts with Microadherence. Clin. Infect. Dis..

[cit54] Pietrocola G., Campoccia D., Motta C., Montanaro L., Arciola C. R., Speziale P. (2022). Colonization and Infection of Indwelling Medical Devices by Staphylococcus aureus with an Emphasis on Orthopedic Implants. Int. J. Mol. Sci..

[cit55] Schairer D. O., Chouake J. S., Nosanchuk J. D., Friedman A. J. (2012). The potential of nitric oxide releasing therapies as antimicrobial agents. Virulence.

[cit56] Silhavy T. J., Kahne D., Walker S. (2010). The Bacterial Cell Envelope. Cold Spring Harbor Perspect. Biol..

[cit57] Gerosa R., Borin M., Menegazzi G., Puttini M., Cavalleri G. (1996). In vitro evaluation of the cytotoxicity of pure eugenol. J. Endod..

[cit58] Dedon P. C., Tannenbaum S. R. (2004). Reactive nitrogen species in the chemical biology of inflammation. Arch. Biochem. Biophys..

[cit59] Wang C., Trudel L. J., Wogan G. N., Deen W. M. (2003). Thresholds of Nitric Oxide-Mediated Toxicity in Human Lymphoblastoid Cells. Chem. Res. Toxicol..

[cit60] Palta S., Saroa R., Palta A. (2014). Overview of the coagulation system. Indian J. Anaesth..

[cit61] Charville G. W., Hetrick E. M., Geer C. B., Schoenfisch M. H. (2008). Reduced bacterial adhesion to fibrinogen-coated substrates via nitric oxide release. Biomaterials.

[cit62] Lantvit S. M., Barrett B. J., Reynolds M. M. (2013). Nitric oxide releasing material adsorbs more fibrinogen. J. Biomed. Mater. Res., Part A.

[cit63] Parker M. C., Shome A., Pinon V. D., Crutchfield N., Garren M., Brisbois E. J., Handa H. (2025). Anti-biofouling organogel and substrate independent coatings with a continuous lubricating network. Chem. Eng. J..

[cit64] Wu Y., Sapkota A., Wilson S. N., Thompson S. G., Garren M. R., Handa H., Brisbois E. J. (2026). Nitric oxide-releasing dextran surface with enhanced albumin affinity mitigates infection and foreign body reaction. Carbohydr. Polym..

