# Supplementary material for: Slippery bioactive nanoemulsion-infused and nitric oxide-releasing surfaces for anti-fouling biomedical applications
Source: Biomater Sci. 2026 Aug 4;14(18):4915–31. doi: 10.1039/d6bm00767h (PMC13448801; doi:10.1039/d6bm00767h)
Supplement: Supplementary file 1 [file BM-014-D6BM00767H-s001.pdf]

Supplementary Information (SI) for Biomaterials Science.
This journal is © The Royal Society of Chemistry 2026

**Slippery Bioactive Nanoemulsion-Infused and Nitric Oxide-Releasing Surfaces for Anti-fouling Biomedical Applications**

# SUPPORTING INFORMATION

Grace H. Nguyen[1], Everett Grizzle[1], Elizabeth J. Brisbois[1]*

[1] School of Chemical, Materials, and Biomedical Engineering, University of Georgia, Athens, GA, 30602, United States

*Corresponding Author

Elizabeth J. Brisbois, Ph.D.

Associate Professor, University of Georgia

302 East Campus Rd.

Athens, GA 30602

Telephone: 706-542-1243

Email: ejbrisbois@uga.edu

## Supporting Tables

***Table S1.*** *Sample names and descriptions.*

| Sample Name | NE Swelled? | Eugenol % | NO Release? |
|---|---|---|---|
| PDMS | - | - | - |
| PDMS-NE | Y | - | - |
| PDMS-2%EG | Y | 2 | - |
| SNAP | - | - | Y |
| SNAP-NE | Y | - | Y |
| SNAP-2%EG | Y | 2 | Y |

***Table S2.*** *Thickness of the samples.*

| Sample Name | Thickness (mm) |
|---|---|
| PDMS | 1.68 ± 0.10 |
| PDMS-NE | 1.99 ± 0.10 |
| PDMS-2%EG | 1.95 ± 0.13 |
| SNAP | 1.62 ± 0.21 |
| SNAP-NE | 1.99 ± 0.21 |
| SNAP-2%EG | 2.02 ± 0.12 |

## Supporting Figures

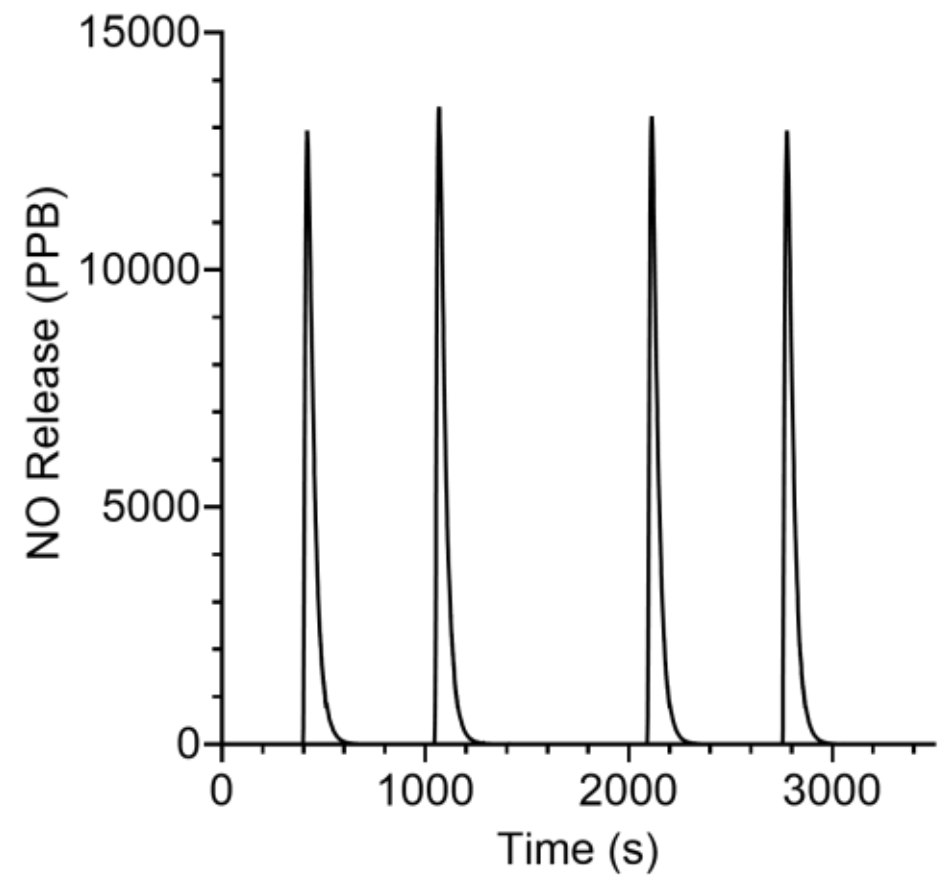


| NO Donor (mg) | PBS (mL) | Injection Volume (μL) | mol NO | Baseline PPB | Corresponding mol NO | Theoretical mol NO | Peak Duration (s) | % Yield |
|---|---|---|---|---|---|---|---|---|
| 3.89 | 4 | 25 | 9.88E-08 | 0.05 | 9.88E-08 | 1.10E-07 | 442 | 89.49 |
| 3.89 | 4 | 25 | 1.00E-07 | 0.05 | 1.00E-07 | 1.10E-07 | 512 | 90.82 |
| 3.89 | 4 | 25 | 9.65E-08 | 0.05 | 9.65E-08 | 1.10E-07 | 654 | 87.42 |
| 3.89 | 4 | 25 | 9.75E-08 | 0.05 | 9.75E-08 | 1.10E-07 | 707 | 88.32 |

***Figure S1.*** *SNAP purity analysis. The area under the curve of each peak was taken to calculate the purity of the SNAP. The purity was averaged and found to be 89.01%.*

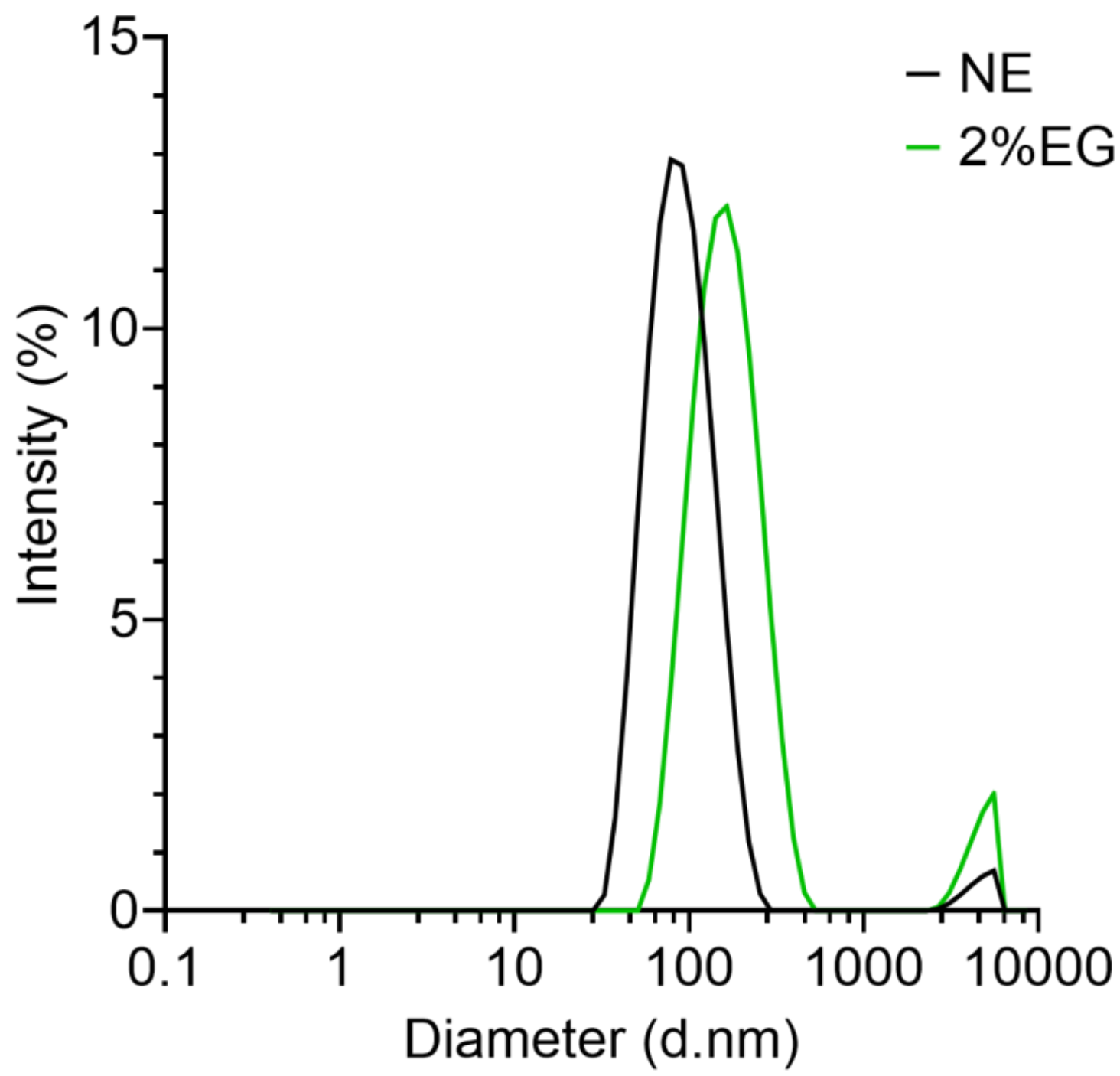


| Sample Name | Average Polydispersity Index (PDI) |
|---|---|
| NE | 0.23 ± 0.03 |
| 2%EG | 0.39 ± 0.03 |

***Figure S2.*** *Hydrodynamic diameters and polydispersity index (PDI) of the plain NE and 2%EG measured using dynamic light scattering. The droplets in the NE had a size of 87.11 ± 3.05 nm while the 2%EG had droplets that were 137.83 ± 6.46 nm (n = 3).*

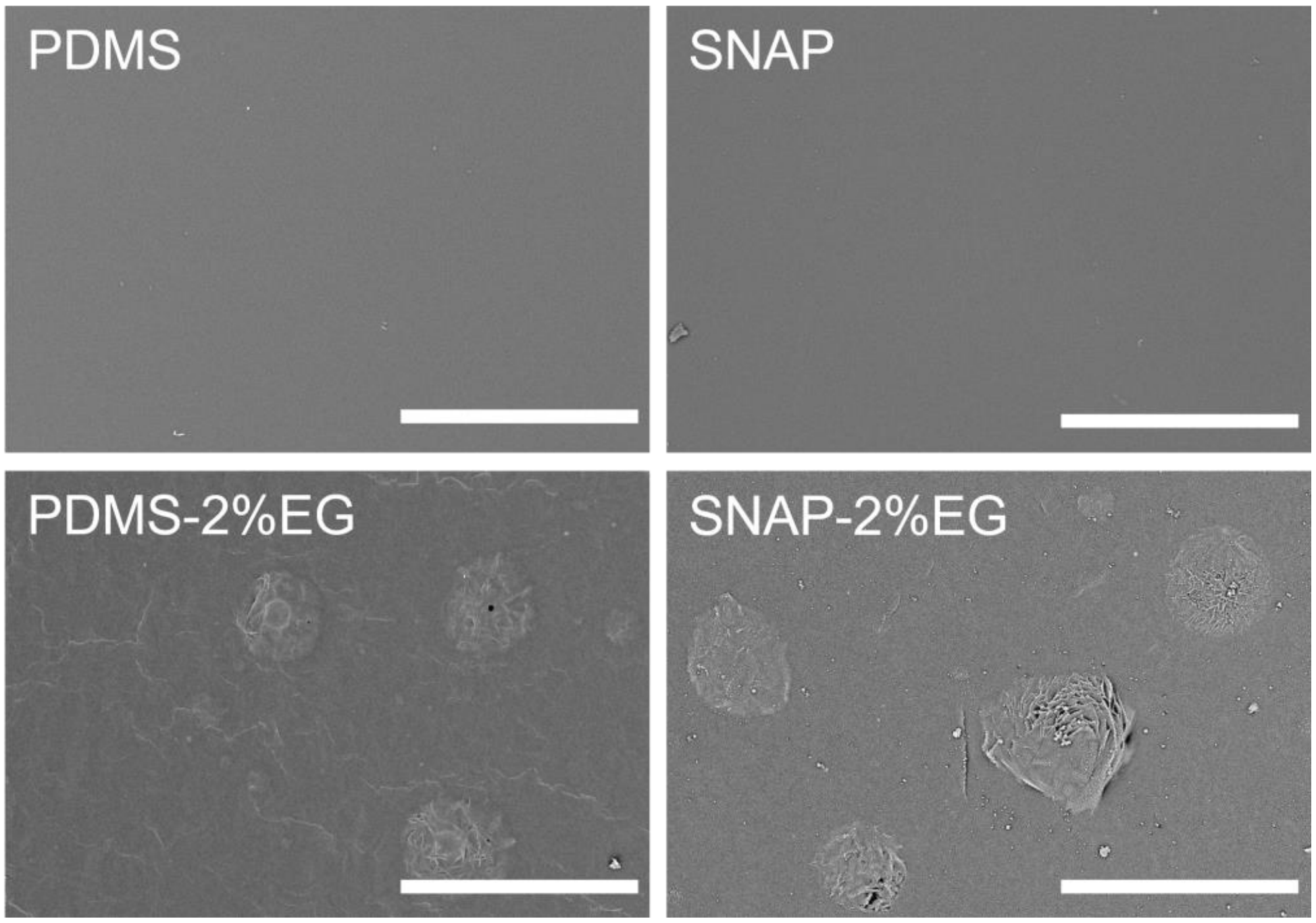


***Figure S3.*** *Representative images of PDMS, SNAP, PDMS-2%EG, and SNAP-2%EG under a cryogenic Scanning Electron Microscope (cryo-SEM). The scale bar represents 200 µm.*

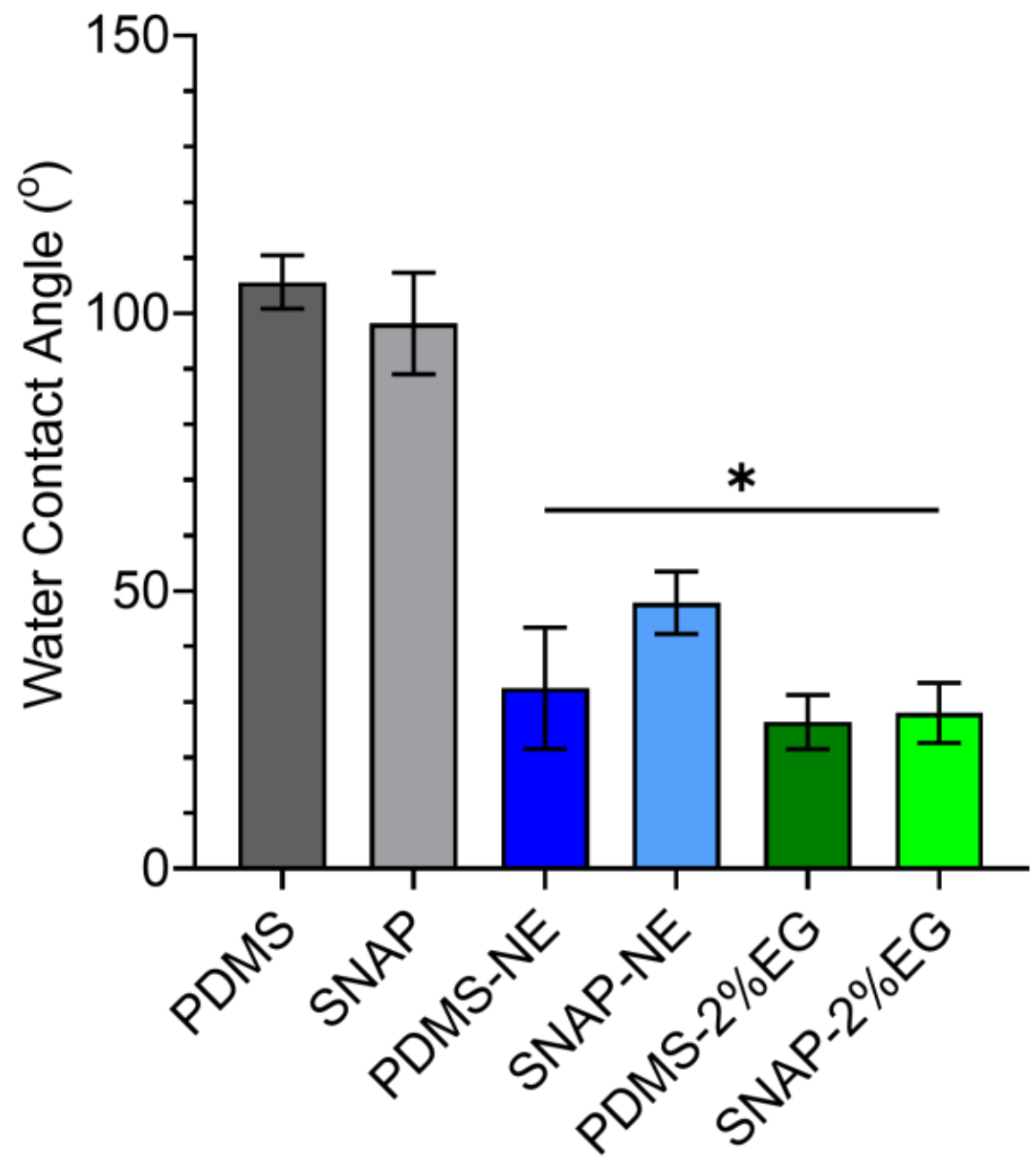


***Figure S4.*** *Static water contact angles of the samples. With the incorporation of the NE into the polymer matrix, the contact angle decreases as the lubricant layer allows the water droplet to spread across the surface (n ≥ 6, * p < 0.05 vs PDMS and SNAP).*

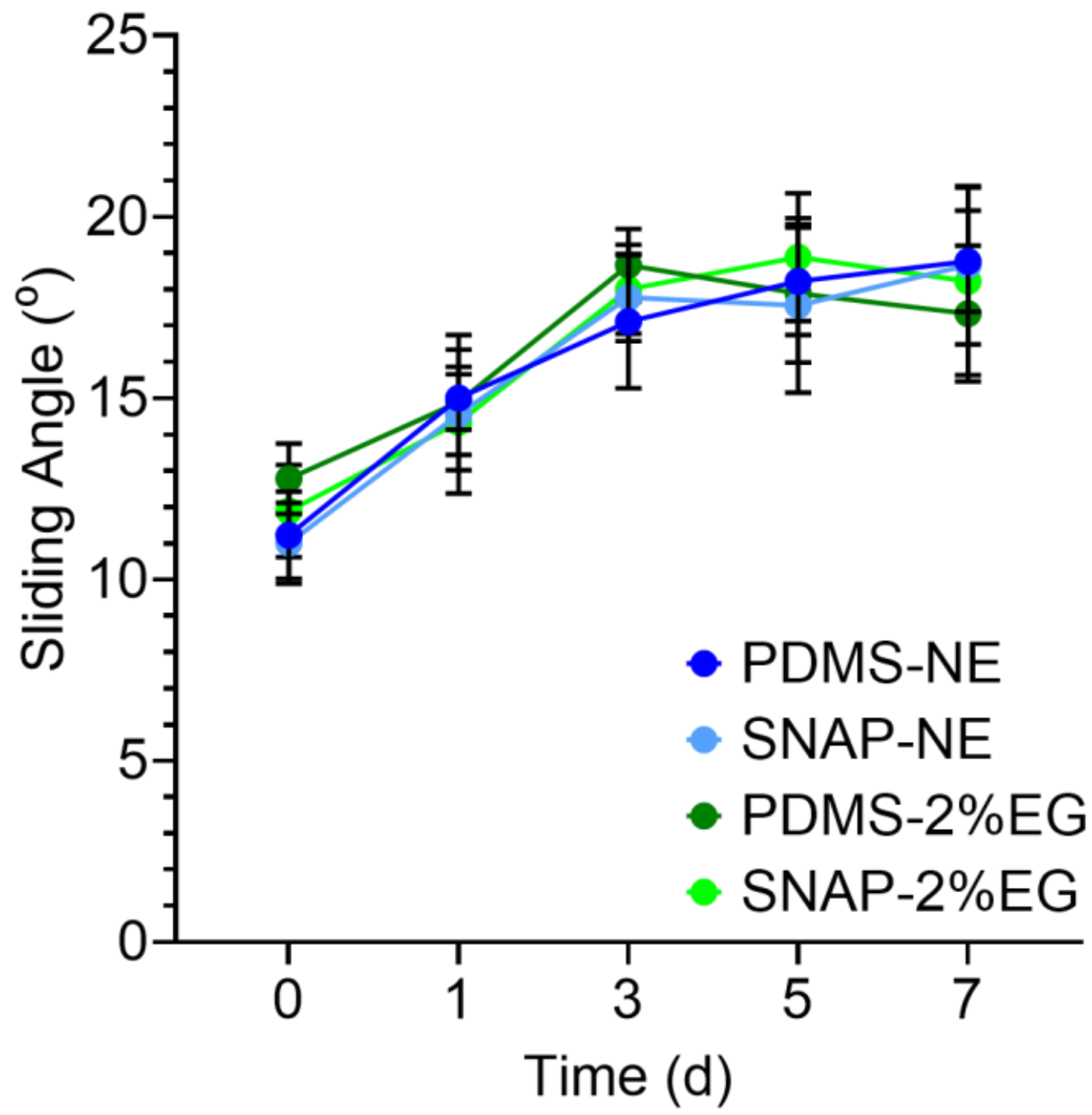


***Figure S5.*** *Sliding angle of the NE-swelled samples over 7 d. Although they increase, the sliding angles of all the sample types remain below 20° for the duration of the study (n = 9).*

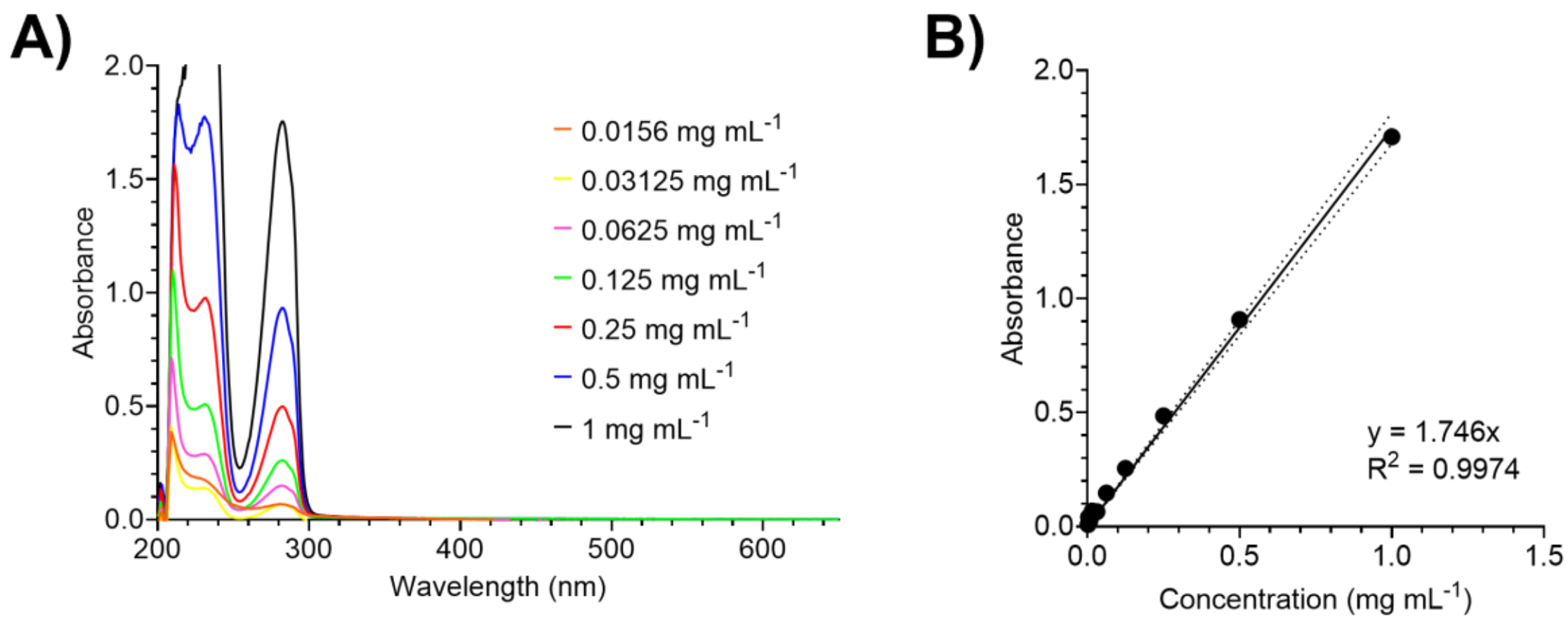


***Figure S6. A)*** *UV-vis spectra of eugenol diluted into THF at different concentrations.* ***B)*** *Standard curve analysis of eugenol in THF ($\lambda_{max}$ = 280 nm).*

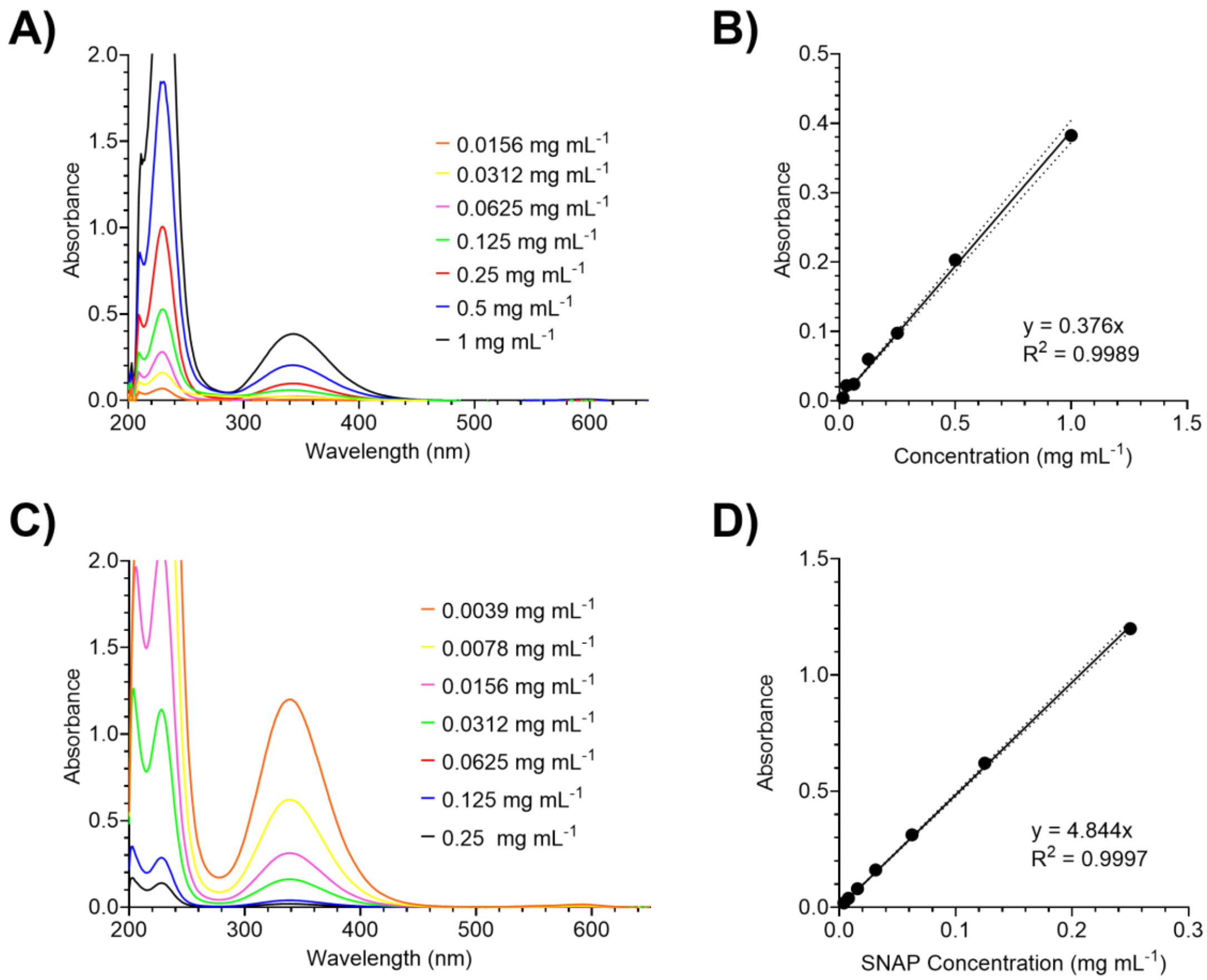


***Figure S7. A)*** *UV-vis spectra of SNAP diluted into THF at different concentrations.* ***B)*** *Standard curve analysis of SNAP in THF ($\lambda_{max}$ = 340 nm).* ***C)*** *UV-vis spectra of SNAP diluted into PBS w/ EDTA at different concentrations.* ***D)*** *Standard curve analysis of SNAP in PBS w/ EDTA ($\lambda_{max}$ = 340 nm).*

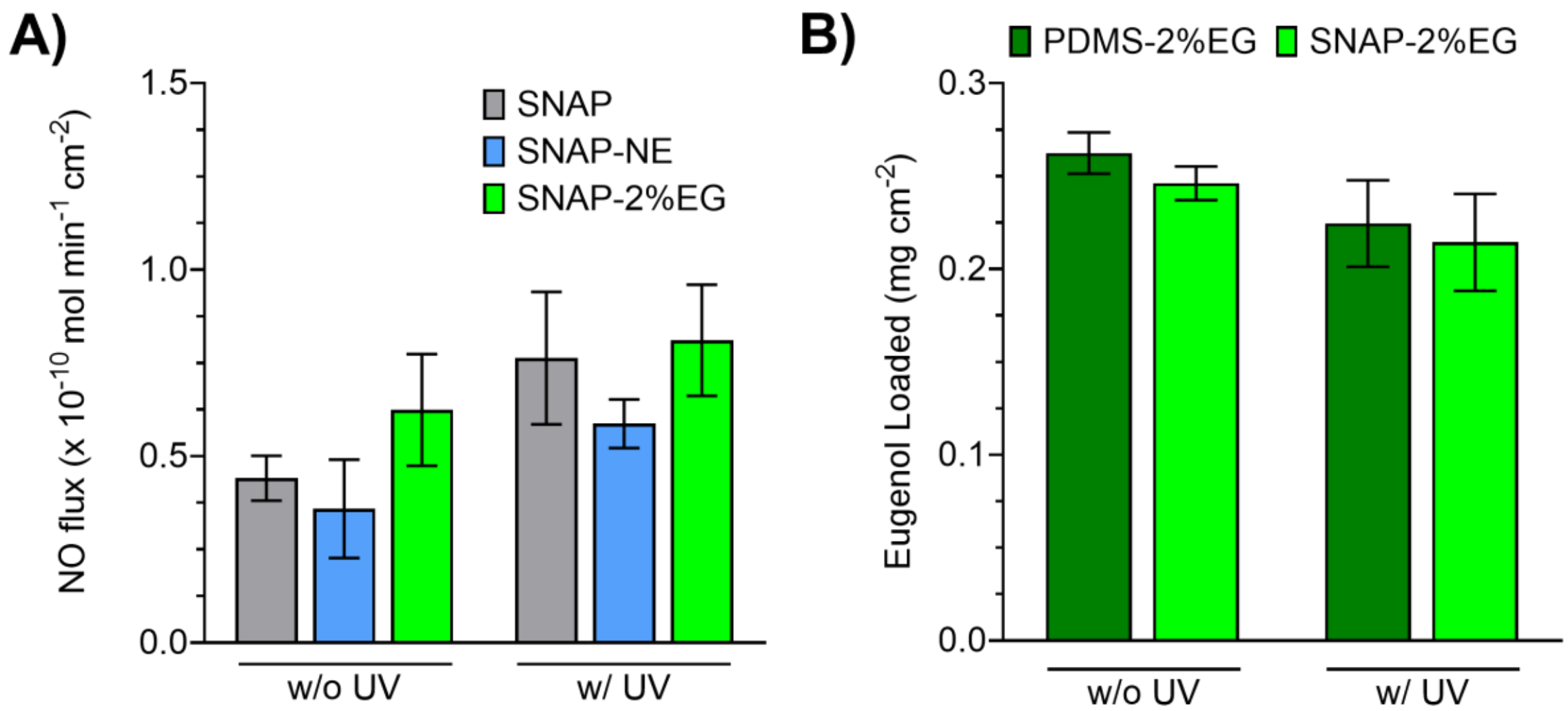


***Figure S8. A)*** *NO release of SNAP-swelled samples before and after UV sterilization. There was no significant difference between the NO released from samples pre-sterilization and post-sterilization ($n = 4$, $p > 0.05$).* ***B)*** *Amount of eugenol loaded into swelled samples before and after UV sterilization. There was no significant difference between the amount of eugenol loaded in the samples pre-sterilization and post-sterilization ($n = 4$, $p > 0.05$).*

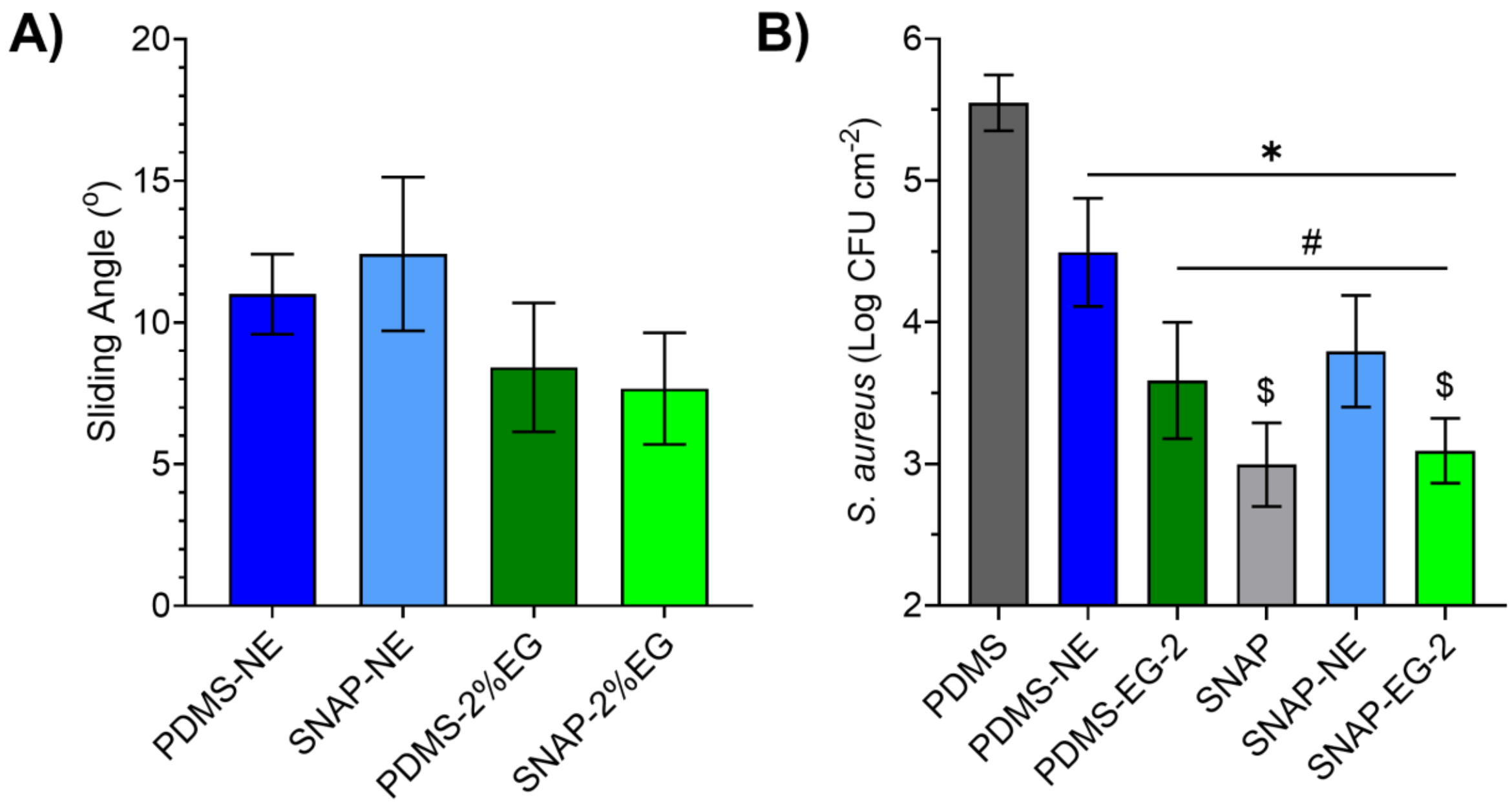


***Figure S9. A)*** *Sliding angle of catheter samples. Although a curved substrate, the catheter surfaces still allowed a water droplet to slide off of the surface and maintain sliding angles below 20° (n = 4, p > 0.05).* ***B)*** *Reduction of bacterial adhesion against S. aureus* on a catheter surface *(n = 4, * p < 0.05 vs PDMS, # p < 0.05 vs PDMS-NE, $ p < 0.05 vs PDMS-2%EG and SNAP-NE). The PDMS-2%EG and SNAP-2%EG samples were able to reduce viable S. aureus by over 2 log.*
